# MethylSense: high accuracy machine learning-based diagnostics for *Aspergillus fumigatus* infection in chickens using host cell-free DNA methylation and Nanopore sequencing

**DOI:** 10.1128/jcm.01054-25

**Published:** 2026-04-27

**Authors:** Markus Hodal Drag, Christina Hvilsom, Louise Ladefoged Poulsen, Henrik Elvang Jensen, Stamatios Alan Tahas, Christoph Leineweber, Carolyn Cray, Mads Frost Bertelsen, Anders Miki Bojesen

**Affiliations:** 1 Copenhagen Zoo 400394, Frederiksberg, Denmark; 2 Department of Veterinary and Animal Sciences, University of Copenhagen, Frederiksberg C, Denmark; 3 Novo Nordisk Foundation Center for Basic Metabolic Research, University of Copenhagen 4321, Copenhagen N, Denmark; 4 Laboklin GmbH & Co. KG 535805 , Bad Kissingen, Germany; 5 Division of Comparative Pathology, Department of Pathology & Laboratory Medicine, University of Miami Miller School of Medicine 12235, Miami, Florida, USA; University of California, Davis, California, USA

**Keywords:** cell-free DNA methylation, Oxford Nanopore sequencing, machine learning diagnostics, avian aspergillosis, host-based biomarkers, epigenetic signatures, fungal infection detection, conservation medicine, liquid biopsy

## Abstract

Avian aspergillosis, caused by *Aspergillus fumigatus* (*Af*), lacks sensitive antemortem diagnostics. Existing microbial cell-free DNA (cfDNA) tests are prone to contamination and require a high pathogen load. We hypothesized that infection-induced tissue damage in chickens creates differentially methylated regions (DMRs) in host cfDNA, enabling machine learning (ML) diagnostics. Serum cfDNA samples (*n* = 124) were obtained from broiler chickens (*n* = 76) with *Af* and non-*Af* infections (*Escherichia coli* or *Gallibacterium anatis*) and controls. Oxford Nanopore sequencing enabled DMR detection and ML training. Performance was evaluated using an independent set (*n* = 49) and 10-repeat Monte Carlo cross-validation (CV) (*n* = 490 evaluations per test) as quality control. A High Accuracy test (93 DMRs, neural network) achieved 98.0% accuracy (sensitivity 95%, specificity 100%, AUC 0.974, PR-AUC 0.928) in the independent set, with CV accuracy 92.0% [95% CI: 89.7%–94.4%]. A Fast test (35 DMRs, SVM) achieved 81.6% accuracy and CV accuracy 79.6% [74.9%–84.3%]. An *In Situ* test (5 DMRs, random forest) designed for field deployment achieved 71.4% accuracy and CV accuracy 62.9% [58.7%–67.0%]. Stratified CV accuracy showed 84.6% [65.1%–95.6%] correct classifications for *E. coli* and 100% [80.5%–100%] for *G. anatis*. Markers showed high bootstrap stability and predominantly overlapped EMARs and enhancers. In conclusion, we present MethylSense (https://github.com/markusdrag/MethylSense), an automated open-source software. The High Accuracy test achieved 92.0% [89.7%–94.4%] CV accuracy (CV sensitivity 94.5% [91.4%–97.6%], CV specificity 90.3% [87.8%–92.9%]). While validated in chickens, MethylSense is adaptable to other species and pathogens, offering scalable, contamination-resilient diagnostics for veterinary and conservation applications.

**IMPORTANCE:**

MethylSense is an automated software for training machine learning diagnostics using differentially methylated regions (DMRs) in cell-free DNA from Oxford Nanopore sequencing. We applied MethylSense to develop three *Aspergillus fumigatus* tests for chickens, each optimized for different clinical scenarios. The High Accuracy test (93 DMRs, neural network) demonstrated 98.0% accuracy, in a blinded test set (*n* = 49) with sensitivity 95%, specificity 100%, ROC-AUC 0.974, and PR-AUC 0.928. Stratified 10-repeat Monte Carlo cross-validation (*n* = 490) showed correct classifications of 84.6% [CI: 65.1%–95.6%] *Escherichia coli* and 100% [80.5%–100%] *Gallibacterium anatis* infected specificity samples. A Fast test for rapid <1 h sequencing (35 DMRs, support vector machine) achieved 81.6% accuracy (sensitivity 80%, specificity 82.8%). An *In Situ* test (5 DMRs, random forest) for field deployment via methylation-specific PCR achieved 71.4% accuracy (sensitivity 45%, specificity 89.7%). Bootstrap analysis demonstrated exceptional marker stability (80.6%–100%) with minimal batch effects, confirming robust host-based diagnostics.

## INTRODUCTION

Avian aspergillosis is a fungal respiratory infection caused by the ubiquitous mold *Aspergillus* fumigatus (*Af*) (1), affecting wild and domestic birds (2, 3). In zoo-housed birds, notably penguins and birds of prey, aspergillosis is a leading cause of morbidity (4–6), while in poultry production, it causes significant economic losses (7). Antemortem diagnosis is usually based on cumulative evidence from clinical observations, fungal culture, endoscopy, diagnostic imaging, as well as hematology, serum protein electrophoresis (SPE), liquid chromatography mass spectrometry (LC-MS) of gliotoxin (8), serum amyloid A (SAA) levels (9), galactomannan and PCR (10), and immunological blotting (11). However, no single technique combines high sensitivity, specificity, cost-effectiveness, minimal invasiveness, and early detection capability.

Circulating cell-free DNA (cfDNA) comprises short (~167 bp) DNA fragments released from apoptotic or necrotic cells into blood and other bodily fluids (12). As the analytical foundation of liquid biopsy (13–15), cfDNA has been investigated as a biomarker for a wide variety of conditions and diseases in mammals: infection (16), mitochondrial (17) and metabolic disorders (18), obesity (19), inflammation (20), physical stress (21), impact of space flight (22), and aging (23). Concentration-based approaches can be difficult to interpret due to natural tissue turnover (24–26), necessitating qualitative interrogation such as nucleosome footprinting (27), fragmentation patterns (28), and methylation patterns (18, 29–31). Oxford Nanopore sequencing (ONT) enables direct measurement of 5-hydroxymethylcytosine and 5-methylcytosine (32–38), enabling identification of tissue-specific differentially methylated regions (DMRs) (18). Previous studies have demonstrated tissue overcontribution of cfDNA during COVID-19 (39), associations between methylation and infection severity (40, 41), and increased cfDNA methylation in active versus latent pulmonary tuberculosis (42).

DNA methylation at cytosine bases represents a dynamic biomarker reflecting disease states and tissue-specific cellular responses. Unlike genetic markers, methylation patterns can change rapidly in response to pathogenic damage, providing a sensitive readout of host-pathogen interactions. Host cfDNA carries tissue-of-origin methylation signatures, enabling non-invasive detection of infection-induced epigenetic changes (32, 35, 36). For fungal infections, where direct pathogen detection is complicated by environmental contamination from ubiquitous organisms such as *Af*, host epigenetic responses may offer a more robust diagnostic signal.

**Fig 1:**
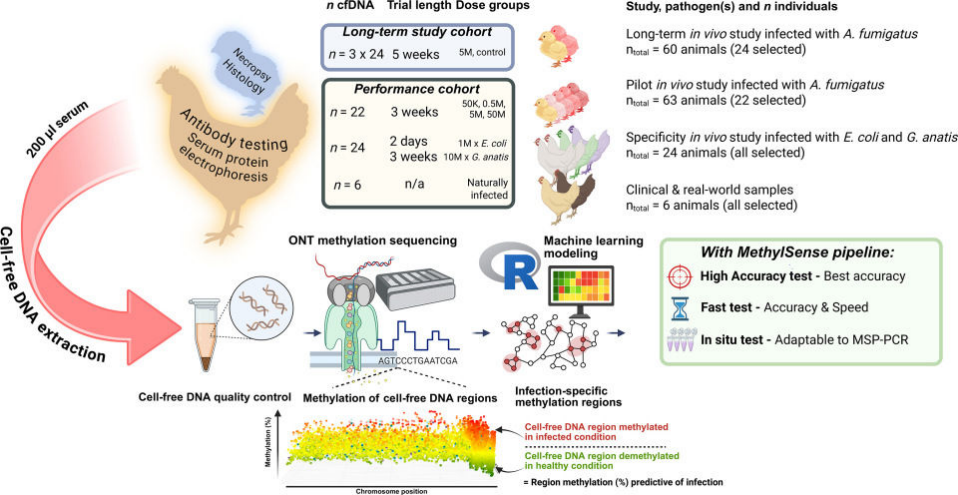
Study design. A total of 124 serum samples from a long-term study (*n* = 72) and performance cohort (*n* = 52) were subjected to cell-free DNA extractions and sequenced with Oxford Nanopore methylation sequencing. Three tests were generated to serve different diagnostic requirements of accuracy, speed, and portability. Created with BioRender.com.

We hypothesized that *Af*-specific DMRs could be identified using ONT sequencing and used to train machine learning (ML) models for infection prediction. The aims were to (i) establish an *in vivo Af* model in chickens and perform ONT sequencing on cfDNA from 200 µL serum; (ii) identify infection-associated DMRs for machine learning classification; (iii) evaluate performance using chickens infected with *Af*, *E. coli,* or *G. anatis*, and naturally exposed birds; (iv) select three diagnostic tests balancing accuracy, turnaround speed, and compatibility with methylation PCR; and (v) develop a reproducible software tool, MethylSense, applicable to *Af* and other pathogens inducing cell death during infection (Fig. 1).

## RESULTS

### Quality control of infection doses by semi-quantitative real-time PCR

Conidial equivalents (CE) from *FKS* copies showed median values of 5.04M (IQR 0.29M) and 4.89M (IQR 0.41M) for the 5M groups in pilot and long-term studies, respectively (non-significant difference). CE values between studies were significantly correlated (Spearman *R* = 0.89, *P* = 0.0014). Full results and *FKS* sequence are in File S1.

### *In vivo* studies for dose determination and model training

**Fig 2:**
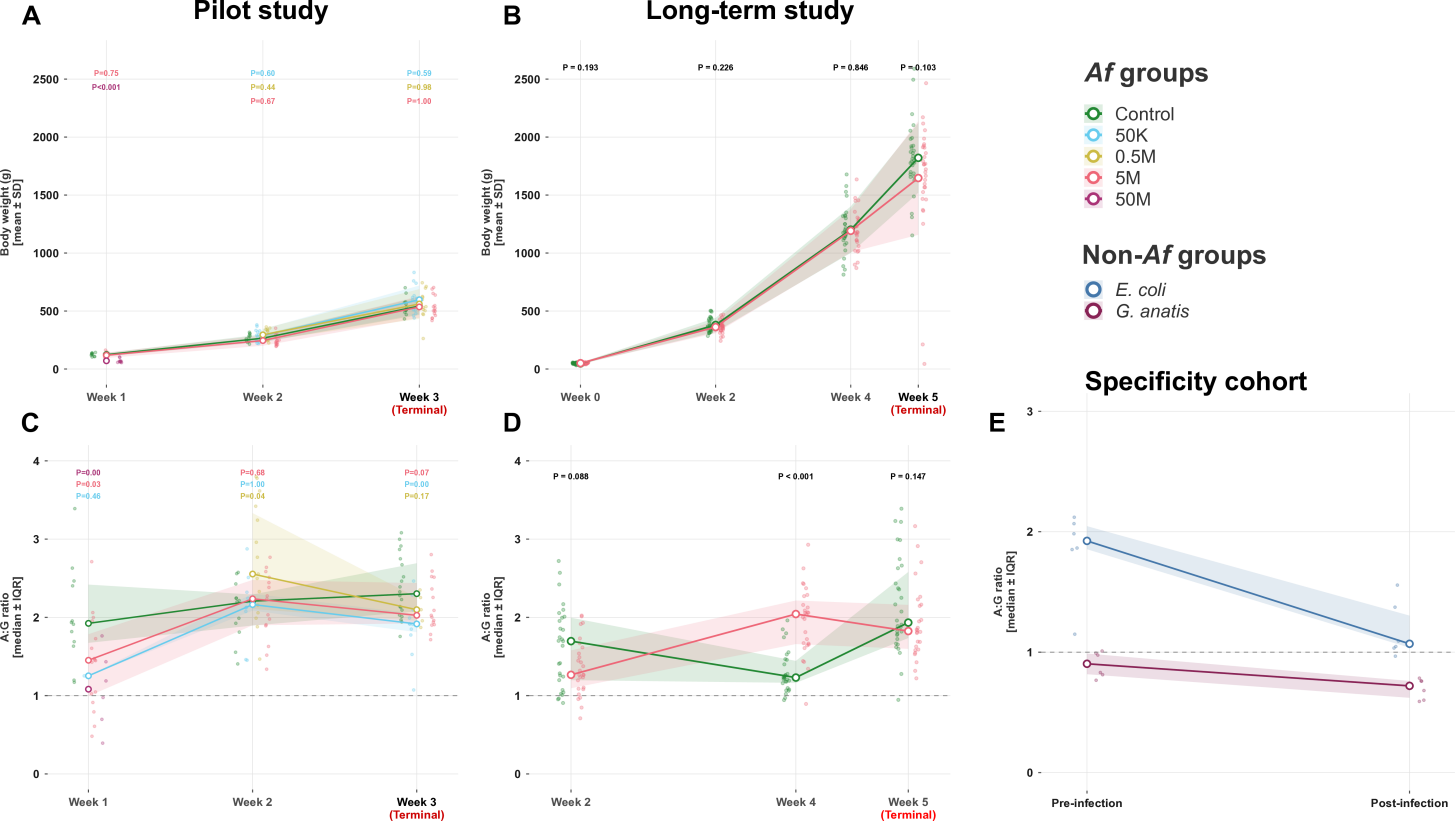
Phenotypic markers during *Aspergillus fumigatus* challenge. (**A**) Body weight in pilot study (*n* = 63); Tukey post hoc *P*-values comparing dose groups to Control at each timepoint; shading indicates mean ± SD. (**B**) Body weight in long-term study (*n* = 60); unpaired t-test *P*-values at each timepoint; shading indicates mean ± SD. (**C**) A:G ratio in pilot study (*n* = 142); pairwise Wilcoxon *P*-values comparing dose groups to Control at each timepoint; shading indicates median ± IQR. (**D**) A:G ratio in long-term study (*n* = 171); Wilcoxon rank-sum *P*-values at each timepoint; shading indicates median ± IQR. (**E**) A:G ratio in specificity cohort (*n* = 24) comparing *Escherichia coli* and *Gallibacterium anatis* infection; Wilcoxon rank-sum *P*-values per pathogen; shading indicates median ± IQR. Dashed horizontal line indicates A:G ratio = 1. Terminal timepoints indicated in red.

In the pilot study, mean body weight decreased significantly (*P* < 0.05) in the 50M group at week 1 (Fig. 2A). Animals in the 50M group were euthanized during days 2–4 due to acute aspergillosis clinical signs. Using cumulative evidence, 5M was selected as an appropriate dose for the long-term study. No significant weight differences were observed in the long-term study between 5M and controls (Fig. 2B).

### Serum protein electrophoresis

A:G ratios decreased significantly (*P* < 0.05) for the 5M and 50M groups (week 1) and for the 50K group (week 3) compared to controls. A tendency to decrease (*P* = 0.07) was also observed for the 5M group in week 3 (Fig. 2C). In the long-term study, A:G ratios had a tendency (*P* = 0.088) of decrease for the 5M group (week 1) but were significantly higher (*P* < 0.05) in the 5M group at week 4 (Fig. 2D). Both *G. anatis* and *E. coli* specificity cohorts showed significant A:G ratio decreases post-infection (Fig. 2E).

### Postmortem examinations, histopathology, and serology-based diagnostics

In the pilot study, 10 scoring parameters differed significantly across groups (FDR < 0.0001), including airsac transparency, lung congestion, and granulomas (File S2A). Histopathology frequencies increased significantly (*P* < 0.05) from 5M (36.7%) to 50M (85%) groups (File S2C and D). No positive *Af*-specific IgG was identified among 67 samples (Table 1). The diagnostic performance of the IgG antibody assay was sensitivity 3.4%, specificity 100%, and accuracy 64.6%. All postmortem scoring parameters are available in File S3.

**TABLE 1:** Diagnostic evaluation of chicken IgG test targeted against *Aspergillus fumigatus* (*Af*) antigens^*a*^

Cohort	*n* samples	TP	FP	TN	FN	Diagnostic accuracy
Long-term study	33	0	0	15	18	45.5%
Pilot study	11	0	0	3	8	27.3%
Specificity cohort— *E. coli* and *G. anatis*	16	0	0	16	0	100%
Clinical and real-world samples	6	0	0	3	3	50%
Positive penguin control	1	1	0	0	0	100%
Total	67	1	0	37	29	64.6%

^*a*^ *n*: number of serum samples tested per cohort; TP: true positive; FP: false positive; TN: true negative; FN: false negative; Af: *Aspergillus fumigatus*.

### ONT sequencing and filtering for *Af*-specific differentially methylated regions

**Fig 3:**
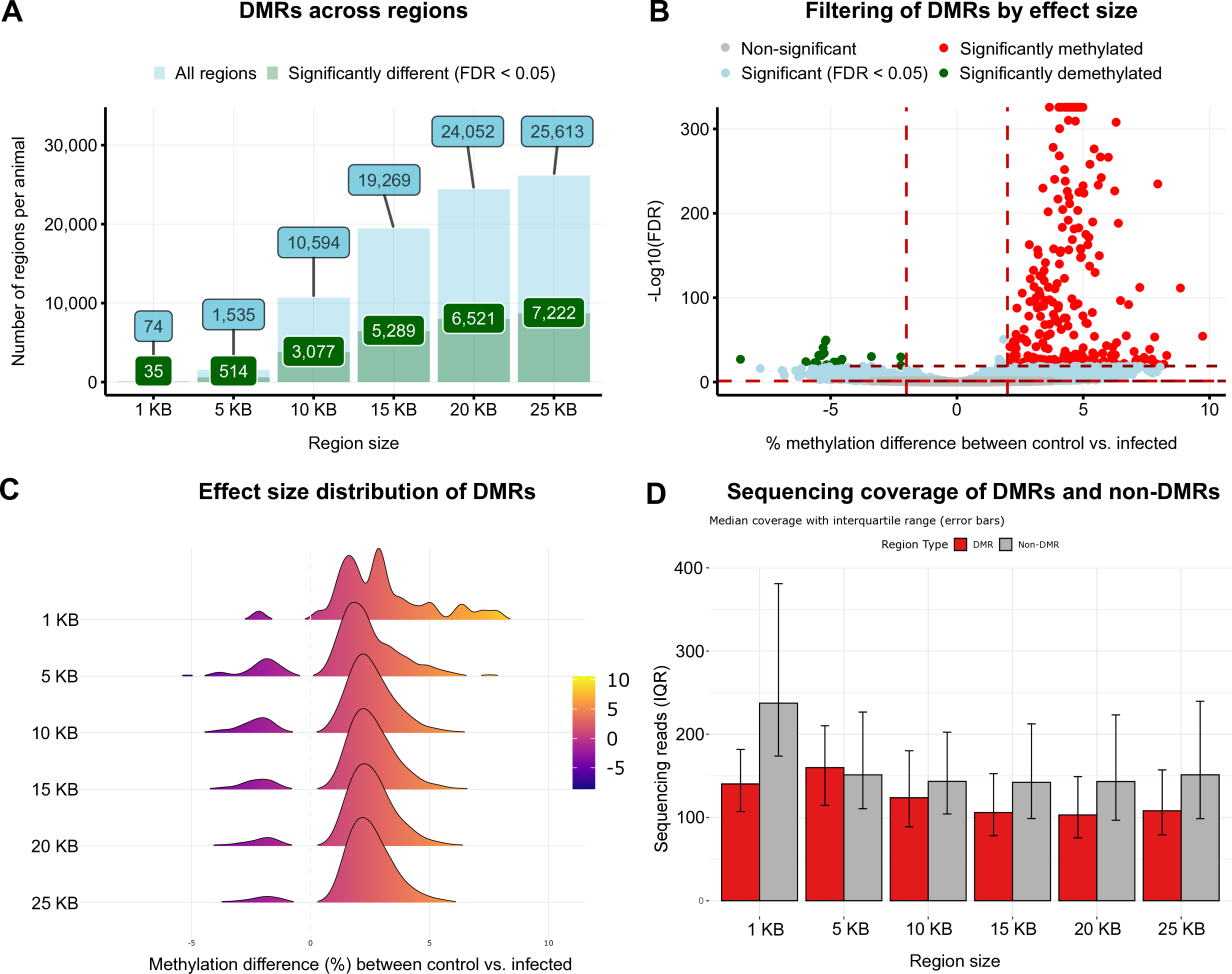
Analyses of differentially methylated regions found in the study. (**A**) DMR detection rates across six region sizes (1–25 KB), showing 47.3% detection at 1 KB and 27.1%–33.5% for larger regions (*n* = 81,137 total methylation regions, 22,658 significant DMRs). (**B**) Effect size volcano plot showing hypermethylation-dominated differential methylation landscape with a mean methylation difference of +3% in infected animals. (**C**) Effect size distribution ridge plots across region sizes, demonstrating consistent hypermethylation patterns: 1 KB (34 hypermethylated DMRs, mean +3.05%), 5 KB (441 DMRs, mean +2.53%), 10 KB (2,681 DMRs, mean +2.60%), 15 KB (4,797 DMRs, mean +2.63%), 20 KB (6,074 DMRs, mean +2.61%), and 25 KB (6,845 DMRs, mean +2.56%). (**D**) Sequencing coverage quality control showing high median coverage across all DMRs: 1 KB (140.3×), 5 KB (159.9×), 10 KB (123.7×), 15 KB (105.9×), 20 KB (103.1×), and 25 KB (108.1×), with overall median 115.9× (IQR 106.5–136.2×).

For 124 samples, the mean cfDNA was 22.3 ng/µL (File S4). Mean sequencing reads were 10.1M and 9.6M for long-term study controls and 5M groups, respectively. A total of 81,137 methylation regions across six region sizes passed the minimum coverage threshold (10×). Of these, 22,658 DMRs were significantly associated with *Af* infection (FDR < 0.05), corresponding to detection rates of 27.1%–47.3% depending on region size (Fig. 3A). The most significant DMRs were hypermethylated in infected animals (approximately +3% mean difference), with relatively few hypomethylated. An effect size filter requiring at least a 5% absolute methylation difference was applied, and only DMRs that passed both statistical and effect size criteria were carried forward for ML model training (Fig. 3B).

### Effect size analysis and sequencing coverage quality control

Across all region sizes (1–25 KB), hypermethylation dominated the differential methylation landscape. Hypermethylated DMRs showed mean effect sizes of +2.53% to +3.05% (medians +2.22% to +2.77%), while hypomethylated DMRs ranged from −2.11% to −2.36% (Fig. 3C). ONT sequencing yielded high coverage across all markers: significant DMRs exhibited median coverage of 103–160× (IQR: 75–210×), regardless of region size, while non-DMR regions showed comparable or higher coverage (142–237× median; Fig. 3D). No samples were excluded as outliers; full statistics are provided in File S5.

### Model selection and performance with independent and cross-validation sets

**Fig 4:**
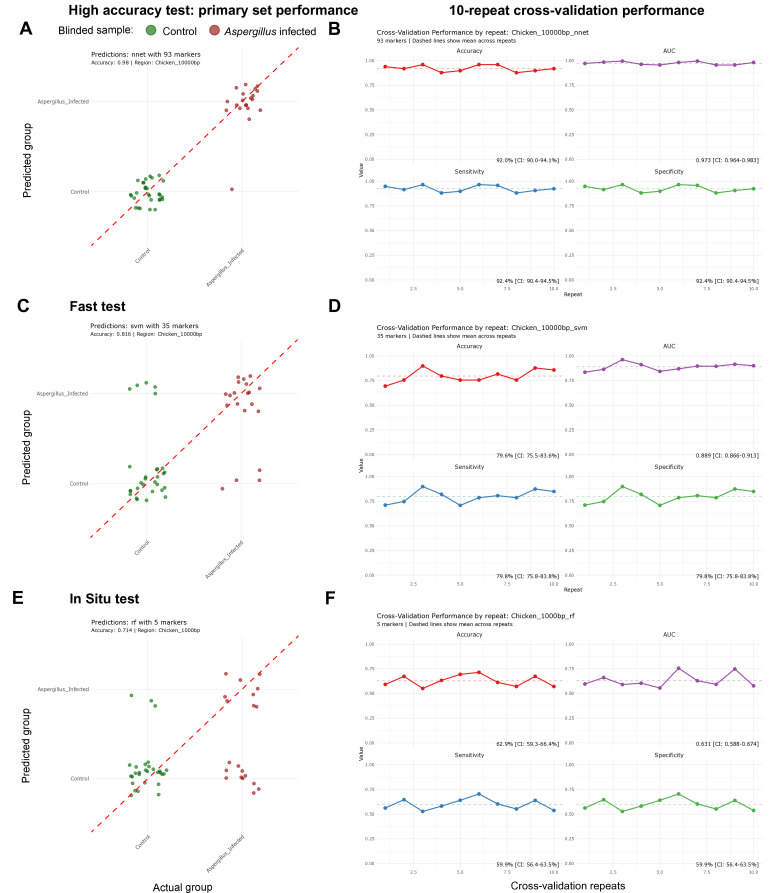
Confusion matrices and performance plots for the three diagnostic tests using the randomized independent test set (*n* = 49, left column) and standard 10-repeat Monte Carlo cross-validation (CV; *n* = 490, right column). Plots (**A**, **C, and E**): Confusion matrices showing first primary model classification results for High Accuracy (98.0% accuracy), Fast (81.6% accuracy), and *In Situ* (71.4% accuracy) tests from the independent test set. Plots (**B**, **D, and F**): Four-panel performance visualization showing performance across 10-repeat Monte Carlo cross-validation; Accuracy, ROC-AUC curves; Sensitivity and specificity curves. All metrics were calculated for *Aspergillus* infection as the positive class.

**Fig 5:**
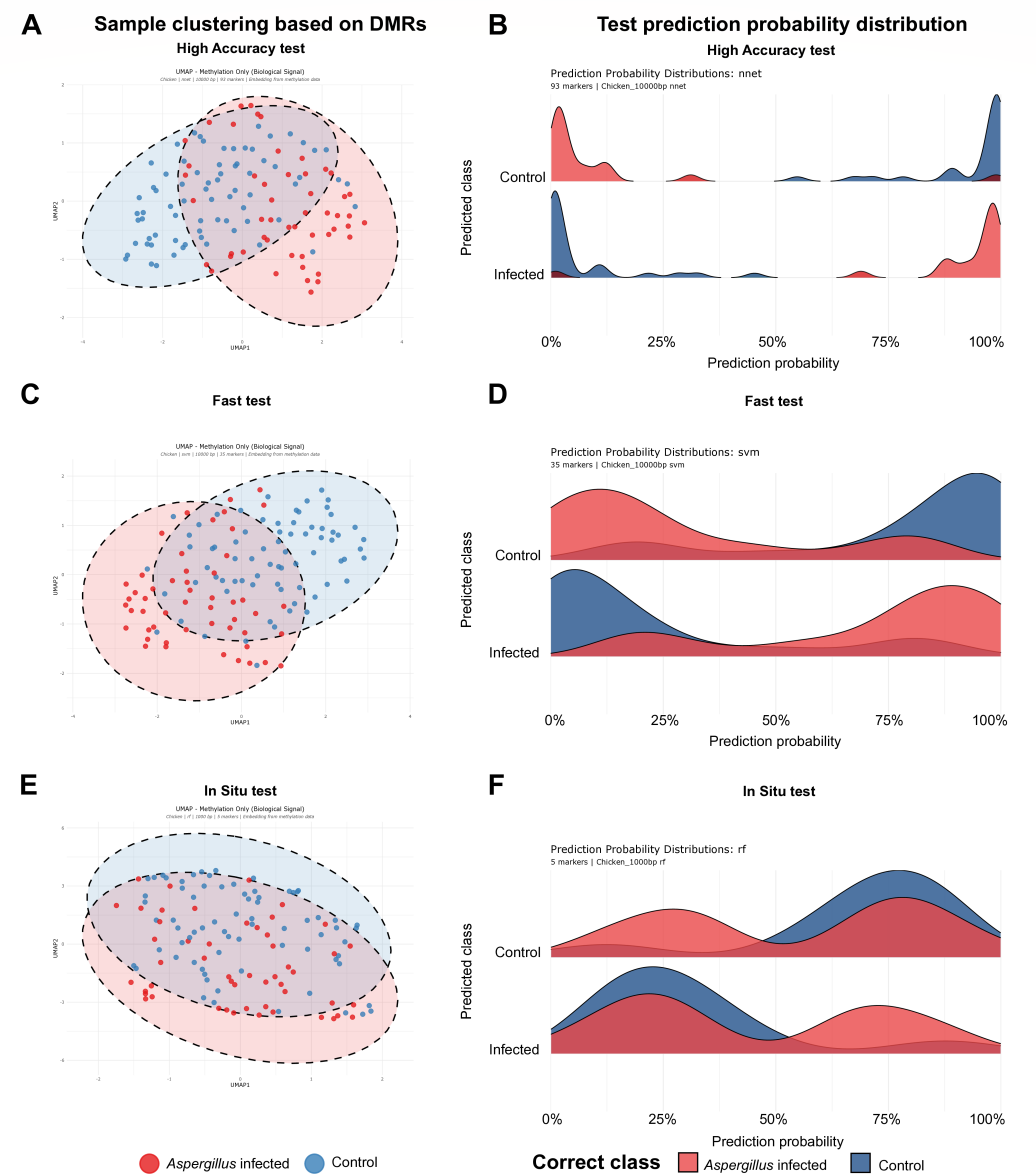
UMAP dimensionality reduction and prediction probability distributions for the three diagnostic tests. Left column from top row: UMAP plots showing sample clustering by infection status (infected vs. control) for (**A**) High Accuracy test (93 DMRs, clear separation), (**B**) Fast test (35 DMRs, moderate separation), and (**C**) *In Situ* test (5 DMRs, limited separation). Right column from top row: Prediction probability distributions showing (**D**) High Accuracy test with mean confidence 94.0% (SD 9.8%), 77.6% of samples >90% confidence; (**E**) Fast test with mean confidence 85.7% (SD 12.8%), 40.8% of samples >90% confidence; (**F**) *In Situ* test with mean confidence 77.1% (SD 10.2%), showing inverse relationship between confidence and correctness. Colors indicate infection status, with clear discrimination in High Accuracy and Fast tests supporting their selection as primary diagnostic tools.

MethylSense screened 394 ML models across six region sizes and seven classification algorithms. Measuring performance on an independent blinded test set (*n* = 49) and via 10-repeat Monte Carlo CV on the full training cohort (*n* = 490), we selected three optimal models. The High Accuracy test (93 DMRs, neural network) was selected for maximum diagnostic performance, achieving 98.0% accuracy (48/49), ROC-AUC 0.974 [0.912–1.000], sensitivity 95%, and specificity 100% in the independent test (Fig. 4A). This high performance was maintained in Monte Carlo CV with CV accuracy 92.0% [CI: 89.7%–94.4%], CV sensitivity 94.5% [CI: 91.4%–97.6%], and CV specificity 90.3% [CI: 87.8%–92.9%] (10 repeats; random 60/40 splits; *n* = 490 evaluations; Fig. 4B; Table 2). Unsupervised UMAP visualization of these 93 DMRs revealed distinct separation between *Af*-infected and control samples, forming two discrete clusters with minimal overlap (Fig. 5A), and the mean (± SD) prediction confidence of the samples was 94.0% (±9.8%) (Fig. 5B).

**TABLE 2:** Diagnostic test performance—independent and Monte Carlo cross-validation test sets^*a*^

Metric	High Accuracy test	Fast test	*In Situ* test
Model specifications			
DMR count	93	35	5
Region size	10 KB	10 KB	1 KB
Algorithm	Neural Network	Support Vector Machine	Random Forest
Independent test set (*n* = 49)			
Accuracy	98.0% (48/49)	81.6% (40/49)	71.4% (35/49)
Sensitivity	95%	80%	45%
Specificity	100%	82.80%	89.70%
PPV	100%	76.20%	75.00%
NPV	96.70%	85.70%	70.30%
ROC-AUC [95% CI]	0.974 [0.912–1.000]	0.898 [0.807–0.967]	0.611 [0.437–0.776]
PR-AUC (infected)	0.928	0.803	0.505
PR-AUC (control)	0.942	0.903	0.64
F1 score	0.974	0.780	0.562
MCC	0.958	0.623	0.396
Mean confidence (SD)	94.0% (9.8%)	85.7% (12.8%)	77.1% (10.2%)
10-repeat Monte Carlo cross-validation (*n* = 490)			
Accuracy [95% CI]	92.0% [89.7%–94.4%]	79.6% [74.9%–84.3%]	62.9% [58.7%–67.0%]
Sensitivity [95% CI]	94.5% [91.4%–97.6%]	81.0% [70.0%–92.0%]	44.0% [36.5%–51.5%]
Specificity [95% CI]	90.3% [87.8%–92.9%]	78.6% [68.9%–88.3%]	75.9% [69.1%–82.6%]
PPV [95% CI]	87.1% [81.9%–91.3%]	72.3% [66.0%–78.1%]	55.7% [47.6%–63.6%]
NPV [95% CI]	96.0% [92.9%–98.0%]	85.7% [80.9%–89.7%]	66.3% [60.9%–71.3%]
ROC-AUC [95% CI]	0.973 [0.962–0.985]	0.889 [0.862–0.916]	0.631 [0.581–0.681]
F1 score	0.919	0.789	0.597
MCC	0.840	0.588	0.209
Odds ratio [95% CI]	160.77 [78.09–330.99]	15.68 [9.98–24.62]	2.47 [1.68–3.64]
Model robustness			
Bootstrap stability	75/93 (80.6%)	34/35 (97.1%)	5/5 (100%)
Temporal dynamics	34/93 (36.6%)	15/35 (42.9%)	0/5 (0%)

^*a*^ All metrics for *Aspergillus* infection detection (positive class). Independent test set: blinded samples withheld from training. Monte Carlo Cross-validation: stratified 10-repeat on *n* = 490 samples. Bootstrap stability: DMRs re-selected with frequency ≥0.75 across 1,000 bootstrap resamples. Temporal dynamics: DMRs showing significant time-dependent methylation changes (Kruskal-Wallis, FDR < 0.05). DMR = differentially methylated region, PPV = positive predictive value, NPV = negative predictive value, MCC = Matthews correlation coefficient.

The Fast test (35 DMRs, SVM) was selected for speed, achieving 81.6% accuracy (40/49), ROC-AUC 0.898 [0.807–0.967], and balanced sensitivity (80%) and specificity (82.8%) in the independent test (Fig. 4C). The Fast test showed CV accuracy 79.6% [CI: 74.9%–84.3%], CV sensitivity 81.0% [CI: 70.0%–92.0%], and CV specificity 78.6% [CI: 68.9%–88.3%] (Fig. 4D). Its corresponding UMAP showed clear segregation of most *Af*-infected samples, though a subset showed partial overlap with the control cluster, reflecting the trade-off in resolution (Fig. 5C), with a mean (±SD) prediction confidence of 85.7% (±12.8%) (Fig. 5D).

The *In Situ* test (5 DMRs, random forest) was selected for field portability, achieving 71.4% accuracy (35/49), ROC-AUC 0.611 [0.437%–0.776] with moderate sensitivity (45%) but high specificity (89.7%) in the independent test (Fig. 4E). The *In Situ* test showed CV accuracy 62.9% [CI: 58.7%–67.0%], CV sensitivity 44.0% [CI: 36.5%–51.5%], and CV specificity 75.9% [CI: 69.1%–82.6%] (Fig. 4F). Consistent with its lower performance, the *In Situ* UMAP displayed moderate clustering with significant overlap between groups (Fig. 5E), and the mean (± SD) prediction confidence was 77.1% (±10.2%) (Fig. 5F).

DMRs from all three tests showed wide multi-chromosomal distribution: the High Accuracy test (93 DMRs) spanned 28 chromosomes; the Fast test (35 DMRs) spanned 17 chromosomes with chromosome 16 being dominant, while the *In Situ* test (5 DMRs) was localized entirely to chromosome 16 (File S6). Study-stratified analyses confirmed that the High Accuracy and Fast tests maintained performance across all cohorts (Table 3), supporting their selection as primary diagnostic tools.

**TABLE 3:** Stratified 10-repeat Monte Carlo CV accuracies across study cohorts^*a*^

Study cohort	Group	*n*_total_	High Accuracy test	Fast test	*In Situ* test
Pilot study	Control	43	81.4% (35) [66.6%–91.6%]	55.8% (24) [39.9%–70.9%]	46.5% (20) [31.2%–62.3%]
	Infected	45	84.4% (38) [70.5%–93.5%]	84.4% (38) [70.5%–93.5%]	26.7% (12) [14.6%–41.9%]
Long-term study	Control	142	88.7% (126) [82.3%–93.4%]	78.9% (112) [71.2%–85.3%]	88.7% (126) [82.3%–93.4%]
	Infected	146	97.3% (142) [93.1%–99.2%]	82.9% (121) [75.8%–88.6%]	52.1% (76) [43.6%–60.4%]
Specificity cohort—*E. coli*	Control	24	100.0% (24) [85.8%–100%]	83.3% (20) [62.6%–95.3%]	83.3% (20) [62.6%–95.3%]
	Infected	26	84.6% (22) [65.1%–95.6%]	65.4% (17) [44.3%–82.8%]	53.8% (14) [33.4%–73.4%]
Specificity cohort—*G. anatis*	Control	23	100.0% (23) [85.2%–100%]	100.0% (23) [85.2%–100%]	87.0% (20) [66.4%–97.2%]
	Infected	17	100.0% (17) [80.5%–100%]	100.0% (17) [80.5%–100%]	76.5% (13) [50.1%–93.2%]
Real-world and clinical samples	Control	15	100.0% (15) [78.2%–100%]	100.0% (15) [78.2%–100%]	46.7% (7) [21.3%–73.4%]
	Infected	9	100.0% (9) [66.4%–100%]	33.3% (3) [7.5%–70.1%]	0.0% (0) [0.0%–33.6%]
TOTAL correct		490	92.0% (451/490) [89.7%–94.4%]	79.6% (390/490) [74.9%–84.3%]	62.9% (308/490) [58.7%–67.0%]

^*a*^ Stratified 10-repeat Monte Carlo cross-validation results (*n* = 490 samples × 3 tests) by study cohort and infection status. Values shown as accuracy (*n* correct) with [95% confidence interval]. DMR = differentially methylated region. *n* = summarized number of randomized samples (but not unique samples) tested in the 10-repeat Monte Carlo cross-validation procedure.

### Sample-level consistencies

Sample-level consistency calculations from the Monte Carlo CV results showed 90.2% (High Accuracy), 70.7% (Fast test), and 47.2% (*In Situ* test) of samples correctly predicted in 80% or more of repeats for High Accuracy, Fast, and *In Situ* tests, respectively. Inconsistent samples were primarily borderline cases (early-stage infections, specificity cohort), with some animals showing persistent ambiguity across timepoints (File S7).

### Nested CV results

Nested CV (10 repetitions, 100 folds, *n* = 1,240 samples) with hyperparameter tuning provided unbiased performance estimates. The High Accuracy test achieved a mean accuracy of 92.9% [91.6%–94.3%], sensitivity 93.5%, and specificity 92.5%; the Fast test achieved 81.3% [79.4%–83.3%], 85.1%, and 78.5%; and the *In Situ* test achieved 68.1% [65.9%–70.2%], 49.4%, and 81.2%, respectively. The high concordance with the standard Monte Carlo CV results validated the model selections.

### Individual DMR importance and effect sizes

DMR contributions were quantified using ANOVA effect sizes (eta squared) and WGCNA (Table 4). In the High Accuracy model, η² values ranged from 0.00 to 0.16: 2 large-effect (η² ≥0.14), 32 medium-effect (0.06–0.14), 49 small-effect (0.01–0.06), and 10 negligible-effect DMRs. The two top DMRs showed 6%–7% methylation differences. The Fast model showed 2 large, 20 medium, and 13 small-effect DMRs. The *In Situ* model showed only small to medium effects (η² 0.03–0.07). WGCNA identified a co-methylated module of 6 DMRs in both High Accuracy and Fast models, including the two highest-effect DMRs (Table 4).

**TABLE 4:** DMR importance and effect sizes^*a*^

Category	High Accuracy test	Fast test	*In Situ* test
Number of DMRs	93	35	5
Effect size distribution (η²)			
Large (η² ≥ 0.14)	2 (2.2%)	2 (5.7%)	0 (0%)
Medium (η² 0.06–0.14)	28 (30.1%)	20 (57.1%)	2 (40.0%)
Small (η² 0.01–0.06)	42 (45.2%)	13 (37.1%)	3 (60.0%)
Negligible (η² < 0.01)	21 (22.6%)	0 (0%)	0 (0%)
Top DMRs			
Highest η²	DMR_1 (0.16)	DMR_1 (0.16)	DMR_3 (0.07)
Second highest η²	DMR_5 (0.15)	DMR_2 (0.15)	DMR_1 (0.06)
Methylation Δ (top DMRs)	6%–7%	6%–7%	4%–5%
Co-methylation modules			
WGCNA modules	One module	One module	–^*b*^
DMRs in the module	6 (DMR_1–5, DMR_7)	6 (DMR_1–5, DMR_7)	–
Module overlap	Shared core module	Shared core module	Too few for WGCNA
Chromosomal distribution			
Chromosomes spanned	28	17	1 (chr16 only)
Top chromosome	Chr16 (9 DMRs)	Chr16 (9 DMRs)	Chr16 (5 DMRs)
Second	Chr2 (8 DMRs)	Chr5 (4 DMRs)	–
Third	Chr5 (8 DMRs)	Chr1,6,17 (3 each)	–

^*a*^ Distribution of effect sizes and co-methylation patterns across diagnostic tests. DMR = differentially methylated region. η² = eta squared (ANOVA effect size). WGCNA = Weighted Gene Co-expression Network Analysis. Methylation Δ = absolute methylation difference between infected and control groups. The shared 6-DMR core module in High Accuracy and Fast tests includes the two highest-effect DMRs, indicating a conserved discriminative signature.

^*b*^ “–” indicates that there were too few DMRs to identify co-methylation modules.

### Batch effects and marker stability

Bootstrap analysis (1,000 resamples) showed high DMR stability: 80.6% (High Accuracy), 97.1% (Fast), and 100% (*In Situ*) of DMRs were re-selected with a frequency of 0.75 or greater (Tables 2 and 5). Linear mixed models testing 13 technical and pre-analytical covariates showed few significant associations after FDR correction. Most technical variables (sequencer, reads, coverage, cfDNA concentration, mapping) showed less than 10% of DMRs with significant associations. Among serum proteins, A:G ratio showed the strongest associations (16%–29% of DMRs). PCA and UMAP confirmed samples did not cluster by technical covariates; infection status remained the dominant source of variance (Fig. 5).

**TABLE 5:** Batch effects and technical confounding analysis^*a*^

Covariate	High Accuracy test	Fast test	*In Situ* test
Sequencing platform			
Sequencer (MinION vs PromethION)	8 (8.6%)	7 (20.0%)	3 (60.0%)
Coverage metrics			
Number of reads	9 (9.7%)	7 (20.0%)	0 (0%)
Mean coverage	4 (4.3%)	4 (11.4%)	0 (0%)
Mapping percentage	0 (0%)	0 (0%)	0 (0%)
Pre-analytical variables			
cfDNA concentration	0 (0%)	1 (2.9%)	3 (60.0%)
Serum protein electrophoresis			
A:G ratio	15 (16.1%)	10 (28.6%)	0 (0%)
Gamma globulin	5 (5.4%)	8 (22.9%)	0 (0%)
Beta globulin	0 (0%)	5 (14.3%)	0 (0%)
Total protein	4 (4.3%)	2 (5.7%)	0 (0%)
Alpha-1 globulin	2 (2.2%)	1 (2.9%)	0 (0%)
Alpha-2 globulin	1 (1.1%)	0 (0%)	0 (0%)
Albumin	1 (1.1%)	1 (2.9%)	0 (0%)
Pre-albumin	0 (0%)	0 (0%)	0 (0%)
Summary			
Total associations	49	46	6
DMRs with ≥1 association	38 (40.9%)	23 (65.7%)	3 (60.0%)
DMRs with no associations	55 (59.1%)	12 (34.3%)	2 (40.0%)
Interpretation	Minimal batch effects	Moderate associations	High platform sensitivity

^*a*^ Linear mixed model (LMM) associations between DMR methylation and technical covariates (after adjusting for infection status). DMR = differentially methylated region. Values shown as the number of DMRs with significant association (FDR < 0.05) and percentage. Despite some technical associations, PCA/UMAP analysis confirmed infection status as the dominant source of variance, and high PR-AUC values indicate biological signal predominance over technical confounding.

### Genomic and epigenomic functional annotations

**Fig 6:**
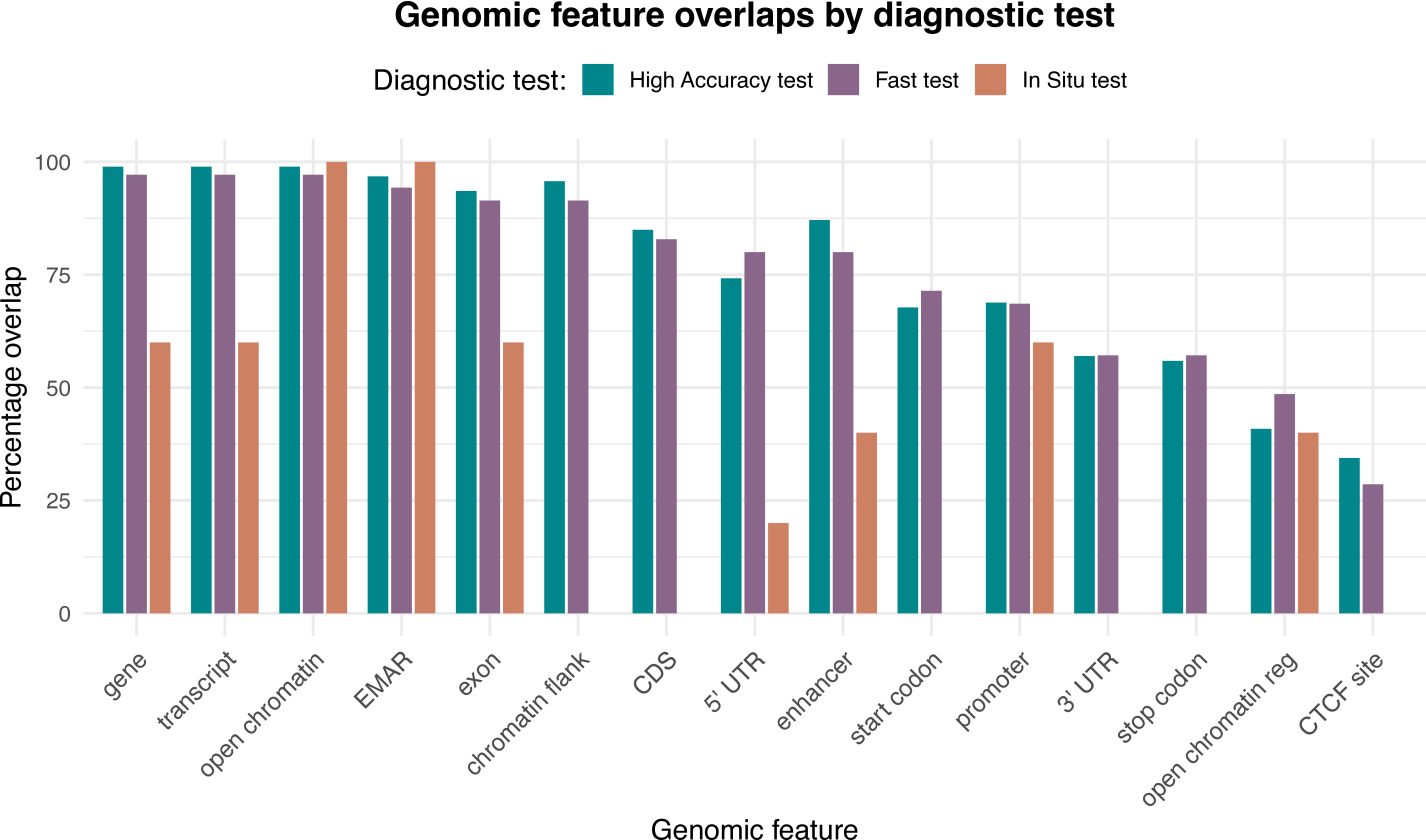
Genomic and epigenomic feature overlap analysis for diagnostic DMRs across the three tests. The grouped barplot shows the percentage of DMRs overlapping 15 genomic feature types for the High Accuracy test (93 DMRs), Fast test (35 DMRs), and In Situ test (5 DMRs). DMRs overlapped preferentially with gene bodies (60.0–98.9%), transcripts (60.0–98.9%), open chromatin regions (97.1–100%), epigenetically modified accessible regions (EMARs, 94.3–100%), and chromatin flanking regions (91.4–95.7%). High overlap was also observed with exons (60.0–93.5%), enhancers (40.0–87.1%), and coding sequences (82.9–84.9%). Moderate overlap was found with 5′UTR (20.0–80.0%), promoters (60.0–68.8%), start codons (67.7–71.4%), 3′UTR (57.0–57.1%), and stop codons (55.9–57.1%). Lower overlap was observed for open chromatin regulatory regions (40.0–48.6%) and CTCF binding sites (28.6–34.4%). The preferential localisation of diagnostic DMRs in regulatory and transcriptionally active genomic regions indicates functional involvement in host response to *Aspergillus* infection rather than random genomic distribution.

DMRs overlapped preferentially with regulatory regions across all tests (Fig. 6). Consistent patterns included: EMARs (94%–100%), gene bodies (60%–99%), transcripts (60%–99%), enhancers (40%–87%), CDS (83%–85%), exons (60%–94%), 5′ UTR (20%–80%), and 3′ UTR (57%–60%). This enrichment indicates DMRs are in chromatin-accessible, transcriptionally active regions, supporting functional roles in host response to *Aspergillus* infection. Median methylation increased significantly (*P* < 0.05) at specific timepoints and in real-world and clinical samples. Methylation at *P* = 0.5 probability threshold was 59.9%, 57.6%, and 70.9% for High Accuracy, Fast, and *In Situ* tests, respectively (Table 6).

**TABLE 6:** Logistic probabilities and median methylation values^*a*^

Probability of infection	High Accuracy test	Fast test	*In Situ* test
*P* = 0.1 (very low)	49.10%	36.40%	20.60%
*P* = 0.25 (low)	54.50%	47.00%	45.80%
*P* = 0.5 (equal)	59.90%	57.60%	70.90%
*P* = 0.75 (high)	65.30%	68.20%	96.00%
*P* = 0.9 (very high)	70.70%	78.80%	121.20%
Methylation range	49.1%–70.7%	36.4%–78.8%	20.6%–121.2%
Dynamic range	21.60%	42.40%	100.60%

^*a*^ Median methylation percentages at key probability thresholds for infection classification. DMR = differentially methylated region. *P* = probability of *Aspergillus* infection. *P *= 0.5 represents the decision threshold where the probability of being infected vs. control is equal. Median methylation values represent the average methylation across all DMRs in each test at the specified probability threshold. The *In Situ* test shows a wider dynamic range than biologically possible due to its minimal 5-marker design.

### Temporal DMR analysis

In the High Accuracy model, 34 DMRs (36.6%) showed significant time-dependent methylation changes (FDR-corrected): 10 increasing, 16 decreasing, and 8 with between-timepoint variation. The Fast test showed 15 DMRs (42.9%) with significant temporal changes. Despite temporal shifts, overall model performance remained stable. *In situ* DMRs showed no significant temporal trends, consistent with its design as a temporally robust signature.

### PCR primer preparation for *in situ* test

Targeted PCR resources were prepared for all five *In Situ* DMRs, including genomic coordinates, UCSC genome browser links, and MethPrimer webserver configurations. Standardized design parameters (primer length 18–27 bp, product size 100–500 bp) enable development of bisulfite PCR, pyrosequencing, or methylation-specific PCR assays as low-plex complements to sequencing-based tests. Full details are available in the model evaluation report in the Zenodo repository.

## DISCUSSION

To our knowledge, we present the first antemortem diagnostic tests for *Aspergillus* fumigatus (*Af*) infection in chickens based on the detection of infection-mediated changes in circulating host cfDNA methylation. Using our novel software MethylSense, we developed three diagnostic tests, where the High Accuracy test (93 DMRs, nnet) achieved up to 98.0% accuracy (sensitivity 95% and specificity of 100%) and impressive robustness (ROC-AUC of 0.974 and PR-AUC of 0.928) on an independent blinded test set. Model evaluation by standard 10-repeat Monte Carlo CV confirmed robust generalization (mean accuracy 92.0% [CI: 89.7%–94.4%], *n* = 490 evaluations), with nested CV providing concordant unbiased estimates (High Accuracy: 92.9% [91.6%–94.3%]; Fast: 81.3% [79.4%–83.3%]; and In Situ: 68.1% [65.9%–70.2%]) indicating performance estimates were not inflated by overfitting.

A major advantage of methylation-based diagnostics is early detection before clinical signs and the wide temporal detection range. For infected pilot study samples taken at week 3 post-infection, the High Accuracy test correctly classified 84.4% [70.5%–93.5%] of the samples in the standard CV evaluations (*n* = 45). For the infected long-term study samples taken at weeks 2, 4, and 5 post-infections, the test correctly classified 97.3% [93.1%–99.2%] of the samples in the standard CV evaluations (*n* = 146) (Table 3). Hence, we confirmed the High Accuracy test could diagnose from 2 to 5 weeks post-exposure, but this could be even earlier or later, pending future validation trials.

Other antemortem diagnostics were inconclusive; only SPE successfully detected significantly lower A:G ratios for the infected groups in the pilot study and in the specificity cohort. However, SPE was also somewhat ambiguous: A:G ratios for the infected group fluctuated compared to controls and were even significantly increased at week 4 in the long-term study. Quality control of SPE data showed inter-assay variability was satisfactory for total protein measurements (CV: 7.3%) but moderate for A:G ratios (CV: 17.9%), which is slightly above the 15% threshold (43). Antibody testing using IgG exhibited only moderate accuracy (64.6%), possibly explained by the chicks being day-old at infection, as critical immune system changes occur in the first 10 days post-hatching when the primary response shifts from Th1 to Th17 (44).

Histopathological assessments confirmed that all tested infected samples exhibited tissue pathology, whereas controls were free of pathology. All 5M and 50M pilot study samples exhibited edema and mononuclear cell presence; 50M samples additionally showed hyphae, granulomas, and giant cells. Pathological lesions were observed in 36.7%–55% of samples for the 5M groups versus 85% (±25.8%) for the 50M group in the pilot study, demonstrating a clear dose-dependency in pathological severity. In the long-term study, some ambiguity was observed with airsac transparency, which scored paradoxically higher in control groups than in infected (mean scores 0.27 vs 0.03) groups. However, both means approached zero, and the difference, while statistically significant, is unlikely to be clinically meaningful.

We hypothesize that the high sensitivity of our tests derives from perturbations to cfDNA methylation from the onset of pathogenesis due to increased infection-mediated cell death. These changes led to altered cfDNA shedding from infected tissues (45, 46) and from dead immune cells (30, 47). High sequencing coverage enabled the resolution of these epigenetic fingerprints to specific pathogens. Clinically, adaptive sampling using a MinION flow cell (48) could analyze thousands of samples with the High Accuracy test, requiring a minimum of 8.3 million bases per sample. Flow cells would be limited to 96 samples due to barcode availability but would remain highly cost-effective. As NGS becomes more prevalent in clinical diagnostics and veterinary/animal sciences exploring the basis of hereditary traits (49) and epigenetic programming (50), ONT sequencing offers an accessible option without requiring specialized staff (51). Alternatively, the *In Situ* test could be adapted to methylation-specific PCR pending rigorous wet-lab validation (52).

A major advantage of analyzing host cfDNA rather than microbial cfDNA (mcfDNA) is freedom from environmental contamination concerns. Host cfDNA comprises 90%–99% of circulating cfDNA, with mcfDNA representing 0.08%–4.85% bacteria, 0.00%–0.01% fungi, and 0.00%–0.16% viral origin (53, 54). M. Kowarsky et al. (55) found that 0.45% of cfDNA reads were nonhuman, with only 1% identifiable in a curated microbiome database, underscoring the difficulty of mcfDNA analysis for early infection detection. This is further complicated by *Af* being ubiquitous (56), which can severely influence mNGS results (57, 58).

Our tests achieve performance comparable to or exceeding existing *Aspergillus* detection methods, which report sensitivities of 53.0%–91.7% and specificities of 71.4%–97.0% using mNGS or PCR on mcfDNA (59–64). The High Accuracy test achieves the upper range of current capabilities. PR-AUC provides more informative metrics than ROC-AUC for imbalanced data sets; the values of 0.928, 0.803, and 0.505 for High Accuracy, Fast, and *In Situ* tests reflect true clinical utility. This is remarkable given that the test detects consequences of infection rather than pathogen DNA. We hypothesize that shifts in cell death dynamics during infection with *Af*, *G. anatis,* or *E. coli* resulted in methylation changes resolvable to causative pathogens.

For the specificity study, 84.6% [65.1%–95.6%] of *E. coli*-infected samples were correctly classified as non-*Af* infected in the CV, whereas this figure was 100% [80.5%–100%] for *G. anatis*-infected samples using the High Accuracy test. The lower specificity against *E. coli* likely reflects shared respiratory pathology, as avian pathogenic *E. coli* frequently causes severe airsacculitis and pneumonia indistinguishable from fungal infections (65), generating overlapping cell death signatures. In contrast, *G. anatis* infections, which often manifest as reproductive tract disorders (oophoritis/salpingitis) (66), appear to generate distinct methylation profiles that are easily distinguished from the *Af* infection changes. Future studies may benefit from adding additional DMRs for better differentiation power.

Bootstrap stability analysis revealed high marker consistency: 80.6% of High Accuracy test DMRs, 97.1% of Fast test DMRs, and 100% of *In Situ* test DMRs were selected in at least 75% of 1,000 bootstrap resamples, indicating genuine biological signals. Linear mixed models testing 13 technical covariates revealed that most were associated with fewer than 15 DMRs after FDR correction. UMAP confirmed samples clustered by infection status rather than technical covariates, supporting the conclusion that performance is driven by biological signal. The *In Situ* test seemed more affected by covariates, highlighting the need for extra validation.

ML-based cfDNA analysis has already debuted in cancer diagnostics (45, 67–69). For infectious diseases, active pulmonary tuberculosis showed higher cfDNA methylation positively correlated with CRP (42), and hypermethylation was observed in COVID-19 patients (40, 41). Our findings were consistent: 97.1% of significant DMRs showed increased methylation with a mean effect size of +3.05%. All five *In Situ* test DMRs localized to chromosome 16, suggesting a specific epigenomic region responsive to *Af* infection. In contrast, High Accuracy test DMRs were distributed across 28 chromosomes, with the highest concentrations on chromosomes 16 (9 DMRs), 2 (8 DMRs), and 5 (8 DMRs). Functional annotation showed 75-95% of DMRs overlapped EMARs, genes, and enhancer regions, suggesting functional roles. Temporal analysis revealed 34 DMRs (36.6%) in the High Accuracy test and 15 DMRs (42.9%) in the Fast test showed significant time-dependent methylation changes, compatible with progressive epigenetic response to infection. None of the five *In Situ* DMRs showed significant temporal trends, consistent with its design as a temporally robust signature. Despite temporal shifts at individual loci, overall model performance remained stable.

The three tests developed in this study offer complementary approaches for different clinical scenarios. The High Accuracy test provides maximum performance for confirmatory diagnosis when sequencing infrastructure is available, delivering up to 98.0% accuracy with 95% sensitivity and 100% specificity in the independent test set results. The Fast test (35 DMRs, SVM) achieves up to 81.6% accuracy with reduced sequencing requirements compatible with less than 1-hour protocols, suitable for screening where rapid results are prioritized. The *In Situ* test (five DMRs, random forest) sacrifices accuracy (71.4%) for experimental simplicity, designed for MSP-PCR or pyrosequencing adaptation without NGS infrastructure. Although sensitivity is lower (45.0%), high specificity (89.7%) makes it suitable as a confirmatory field test where false negatives may occur but positive results can be trusted.

Finally, sample consistency analysis showed that 90.2% and 70.7% of samples were predicted correctly in at least 80% of standard CV sub-sampling repeats for High Accuracy and Fast tests, respectively, versus 47.2% for the *In Situ* test. Inconsistent samples primarily belonged to challenging cohorts such as early-stage infections and specificity cohorts, suggesting residual errors arise from biologically ambiguous methylation profiles rather than model instability.

### Cost-effectiveness considerations

Estimated per-sample costs for the High Accuracy test are approximately $50–100 when amortizing a MinION flow cell over 96 samples (including library preparation and reagents), while the Fast test costs approximately $30–60 with a turnaround of 30–45 min with adaptive sampling or 45–60 min without, due to reduced sequencing requirements (48). The projected *In Situ* test, once validated, would cost approximately $5–15 per sample using MSP-PCR reagents (70). In comparison, existing diagnostics include galactomannan ELISA ($30–80) (71), culture-based methods ($50–100, requiring necropsy) (72).

While ONT-based tests may have higher per-sample costs, several factors offset these: early detection reduces treatment costs and mortality; resistance to environmental contamination reduces repeat testing; and multiplexing allows 96 samples per flow cell. We acknowledge that formal cost-effectiveness modeling incorporating treatment costs, mortality rates, and QALYs would be valuable future work. The workflow was compiled into MethylSense (https://github.com/markusdrag/MethylSense), enabling reproducible analysis and adaptation to new pathogens. MethylSense provides automated DMR detection, ML model training with nested CV, bootstrap stability analysis, batch effects testing, and comprehensive reporting.

### Limitations

Sample sizes for real-world and clinical samples were limited, making it difficult to assess true breed or age bias. In this study, chickens were of varied ages, and, at the very least, three breeds were represented. Although the independent test set (*n* = 49) provided robust performance estimates as confirmed by standard and nested CV, larger validation cohorts are necessary before routine clinical implementation. To confirm standard CV sensitivity and specificity for each test, Cochran’s formula for precision (at 95% confidence level) calculates that a future validation trial should include 228, 500, and 718 positive and negative samples, respectively. In future trials, the temporal stability of methylation markers over extended infection periods (>5 weeks) would be an obvious research question to include as well. Longitudinal monitoring could enable better treatment response assessment and prognosis. Another limitation of the current study is the influence of technical covariates in the *In Situ* test, where 60% of DMRs were significantly associated with the choice of sequencer and cfDNA concentration. For future use, especially in field deployment, this should be addressed through standardized laboratory workflows to ensure high analytical consistency.

It would also be interesting to analyze the relationship between methylation changes and other biomarkers. For instance, recent predictive modeling in aquatic birds demonstrated that elevated 3-hydroxybutyrate and beta-globulins, combined with respiratory clinical signs, could predict aspergillosis with high performance (AUC 0.98) (73). Moreover, exploring acute phase proteins like SAA and AGP (74) and methylation could be interesting, given that *Af* can induce acute inflammatory responses through SAA stimulation (9, 74). Integrating metabolic and proteomic biomarkers with cfDNA methylation could potentially enhance diagnostic precision.

The limited curation of the chicken genome precluded statistical functional overrepresentation tests using the DMR coordinates. The biological mechanisms linking *Af* infection to specific methylation changes in cell types still remain unknown due to the lack of a universal chicken methylation atlas. With ONT sequencing data from this study now available, further work is encouraged to deconvolute the cfDNA to their tissue of origin when appropriate references become available. Finally, adaptation of the tests to other bird species is highly attractive but would probably require a complete repetition of the current study, from data collection to model discovery and validation in MethylSense for successful translation.

### Conclusion

The High Accuracy test demonstrated both excellent independent test set and CV results (CV accuracy 92.0% [CI: 89.7%–94.4%], CV sensitivity 94.5% [91.4%–97.6%], CV specificity 90.3% [87.8%–92.9%]), as well as high model robustness. Thus, we demonstrated that MethylSense can successfully train accurate diagnostic models for detecting *Af* infection using ONT methylation sequencing of small (200 µL) volumes of serum cfDNA. The performance was equal to or better than current tests detecting *Af* using mNGS and PCR on mcfDNA, with complete freedom from environmental spore contamination concerns. The Fast test demonstrated rapid screening capability with acceptable CV diagnostic accuracy (79.6% [74.9%–84.3%]), while the *In Situ* test (5 DMRs, chromosome 16) showed a simplified PCR-compatible (1KB DMRs) alternative pending future wet-lab and limit of detection (LOD) validation. These pre-trained models are publicly available in our repository, enabling the prediction of infection status in new, unknown samples following ONT sequencing of host cfDNA and using the MethylSense predict function. Currently developed for chicken, the tests have major potential for adaptation to endangered avian species for conservation health monitoring. As the approach can be trained to recognize infection in any host with circulating cfDNA, MethylSense has major potential for translation to early human invasive aspergillosis detection in immunocompromised patients.

## MATERIALS AND METHODS

### Animal model overview

A full overview of the study design is shown in Fig. 1. Briefly, a total of 124 cfDNA samples from a total of 76 individual chickens were sequenced and used in this study. The samples were comprised of multiple *in vivo* studies as well as clinical and real-world samples: (i) a long-term *in vivo* study infected with *Af* (*n*_total_ = 60 chickens), where a subset of 24 chickens were sampled for sequencing at three timepoints (weeks 2, 4, and 5) producing 72 sequenced cfDNA samples, (ii) a pilot *in vivo* study (*n*_total_ = 63 chickens) where 22 cfDNA samples were sequenced from 22 chickens, (iii) specificity *in vivo* cohorts (*n* = 24 chickens) comprising 24 equally divided case and control *E. coli* and *G. anatis* sequenced cfDNA samples, and (iv) clinical and real-world samples (*n* = 6 animals) with 6 sequenced cfDNA samples. The total number of sequenced cfDNA samples (*n* = 124) was pooled and randomly split into a training set (*n* = 75) and an independent blinded test set (*n* = 49) for ML model development and evaluation, respectively. The randomized split ensured that samples from any study could be assigned to either set, preventing study-specific bias in model training and evaluation.

Practically, the experimental animals (*n*_total_ = 123 individuals) used in the pilot and long-term *in vivo* studies before sequencing selection were commercial day-old hybrid broiler chickens (Ross-308) procured from DanHatch A/S and housed at the Department of Veterinary and Animal Sciences, in a Biosafety Level 2 facility (Frederiksberg, Copenhagen, Denmark). Upon arrival, the chicks acclimatized for 1 day before initiation of the study, and throughout the two models, all chickens were provided with *ad libitum* broiler feed, fresh water, sand, and adequate heating. The temperature was monitored and kept consistently at 25°C (±0.5°C), and daily care was provided by animal caretakers. All animal experiments were approved by the National Danish Animal Experiments Inspectorate (Dyreforsøgstilsynet), license number (2019-15-0201-01611) and the Copenhagen Zoo Animal Care and Use Committee (1 October 2022). All animal experiments detailed in this study comply with the Animal Research: Reporting of *In Vivo* Experiments (ARRIVE) guidelines and EU Directive 2010/63/EU for animal experiments.

### Mycological cultures and preparation of inoculum

Stock culture of *Af* strain CBS112.33 was kindly provided by Centraalbureau voor Schimmelculture (CBS) (Utrecht, NL). Conidia were obtained and prepared by protocol from S. Thierry et al. (75) with slight modifications. Stock culture was plated under sterile conditions on Sabouraud dextrose agar (SDA) plates and incubated at 25°C for 10 days. After incubation, the plates were transferred to a sterile fume hood for conidia isolation. Conidia from each plate were harvested by sequential flushing with a total of 10 mL sterile PBS containing 0.01% (vol/vol) Tween-20, which was collected into 15 mL Falcon tubes and transferred directly to microscopy facilities. Subsequently, 10 µL gently inverted suspension from each tube was carefully counted in a Fuchs-Rosenthal counting chamber by light microscopy at 400× magnification.

Tubes with the enumerated conidia were pelleted by centrifugation at 4,000 × *g* for 30 min at 4°C, supernatant discarded, and finally, the volumes were collected and resuspended to a master suspension of 5 × 10⁸ conidia in 10 mL sterile PBS. The master suspension was used to create a dilution series with dose concentrations of 5 × 10⁷, 5 × 10⁶, 5 × 10⁵, and 5 × 10⁴ conidia in a 100 µL volume, from now on designated 50M, 5M, 0.5M, and 50K. These four final suspensions were stored at 4°C and used as inoculum for experimental animal inoculations. To ensure the quality of the conidia, the whole procedure was always conducted the day before the start of a new animal experiment.

### Quality control of infection doses by semi-quantitative real-time PCR

To determine the consistency and accuracy of the two inocula created for the pilot study and the long-term study, as well as ensuring the dose concentrations, a TaqMan probe-based real-time quantitative PCR (qPCR) assay was used in conjunction with plasmid DNA to accurately assess the conidial equivalents (76) following the procedure by E. Melloul et al. (77). This procedure takes advantage of the *FKS* gene being a single gene in the *Af* genome. As conidia are haploid units (78) and thus only carry a single FKS copy, the number of conidia can be determined by a semi-quantitative standard curve using plasmid DNA with known molarities. Briefly, 100 µL of the prepared inocula corresponding to an individual chicken dose was used as input material for the Qiagen DNeasy Blood & Tissue kit (Qiagen, Hilden, GmbH) following the manufacturer’s instructions for a total elution volume of 200 µL. Genomic DNA (gDNA) was quantified using a NanoDrop One UV/Vis spectrophotometer (ThermoFischer, Denmark). Conidial equivalents were quantified by primers and probes for the single-copy *FKS* gene, previously reported by C. Costa et al. (79): Sense amplification primer, AFKS1 (5′-GCCTGGTAGTGAAGCTGAGCGT-3′), antisense amplification primer, AFKS2 (5′-CGGTGA ATGTAGGCATGTTGTCC-3′), and the TaqMan FKS probe (5′−6-FAM-TCACTCTCTACCCCCATGCCCGAGCC-BHQ1-3′). For semi-quantification by standard curve, we constructed a 490 bp long synthetic DNA fragment with known molarity of the *FKS* gene (GenBank accession: U79728, from bp 2,731 to bp 3211, File S1), which was procured from Invitrogen GeneArt Synthesis (ThermoFisher, Denmark) and diluted into five log₁₀ concentrations. The final reaction mixtures were as follows: 2 µL DNA, 0.9 µM sense and antisense primers, 0.2 µM FKS TaqMan probe, 10 µL TaqMan Fast Advanced Master Mix for qPCR (Applied Biosystems, Roskilde, Denmark), and 4 µL nuclease-free H₂O in 20 µL reaction volume. The qPCRs were run using a LightCycler 96 detection system (Roche, Meylan, France) under the following conditions: 50°C for 2 min, 95°C for 10 min, then 50 cycles of 15 s at 95°C and 1 min at 65°C (77). All data were analyzed with LightCycler 96 Application Software (v. 1.1.0.1320, Roche, Switzerland) and exported to Microsoft Excel. Amplification efficiency was calculated as:


$$E=10^{-1/\text{slope}}$$

where the slope is derived from the standard curve of cycle threshold (Ct) values versus log₁₀ of template concentration. All qPCR experiments were reported in accordance with the MIQE guidelines (80). Finally, median (± IQR) *FKS* gene copy counts were compared statistically with the enumerated microscopy counts and between similar dose concentrations between the two *in vivo* studies using a Kruskal-Wallis test.

### *In vivo* studies: pilot study for *Af* dose determination

A total of 63 one-day-old chickens divided into five groups comprised a pilot study to determine the appropriate dose of the *Af* inoculum for the later long-term study. After 1 day of acclimatization, each of the five groups was subjected to intra-tracheal infection of 100 µL PBS containing 50K, 0.5M, 5M, or 50M conidia or only sterile PBS. Monitoring of the animals was done intensively, with hourly visual health and behavioral checks for the first 3 days with the exception of 8 h nightly breaks. In accordance with the Danish Animal Experiments Inspectorate guidelines (Danish Animal Experiments Inspectorate 2025), a maximum of 1.5 mL blood was drawn for serum protein electrophoresis (SPE) and cfDNA sequencing from the brachial vein on day 8 (week 1), 15 (week 2), and 23 (week 3, trial termination). Blood was collected into standard 1.5 mL Eppendorf tubes. Blood from four additional eggs served as extra negative controls before the pilot study trial started, from which blood was drawn directly through the shell one day before hatching prior to euthanasia by rapid cervical dislocation. Serum was obtained by letting blood samples coagulate overnight at room temperature and subsequent centrifugation at 2,000 × *g* for 15 min at 4°C. All serum samples were stored immediately at −80°C after collection and processing. After the trial termination, postmortem examinations combined with daily clinical observations, serum protein electrophoresis (SPE) data, and histopathology were used to determine the appropriate dose for the subsequent long-term study.

### *In vivo* studies: long-term study for model training

A total of 60 one-day-old chicks were divided into two equally sized groups and housed in separate rooms in temperature- and humidity-controlled isolation facilities. After 1 day of acclimatization, all chickens were dosed intra-tracheally with 100 µL sterile PBS containing either 5M conidia (as determined in the pilot study) or only sterile PBS. Blood for SPE and cfDNA sequencing was drawn from the brachial vein on day 15 (week 2), day 29 (week 4), and day 35 (week 5, trial termination), and all animals were weighed. Blood was collected into standard 1.5 mL Eppendorf tubes. Serum was obtained by letting blood samples coagulate overnight at room temperature and subsequent centrifugation at 2,000 × *g* for 15 min at 4°C. All serum samples were stored immediately at −80°C after collection and processing.

### Postmortem gross examination

Euthanasia was conducted by rapid cervical dislocation at trial termination after weighing and blood collection. To assess pathological changes in the respiratory tissues and organs, necropsies of each individual bird were conducted within 1 h of euthanasia. All animals from the pilot study (*n* = 63) were subjected to postmortem examinations, comprising the groups 50K (*n* = 13), 0.5M (*n* = 13), 5M (*n* = 15), 50M (*n* = 10), and the control group (*n* = 12).

For the long-term study, all animals (*n* = 60) were subjected to postmortem examinations, comprising the group 5M (*n* = 30) and the control group (*n* = 30). A pre-developed necropsy scoring sheet was used to evaluate the peritoneum, left airsac, left lung (with weight), spleen (with weight), liver, and trachea. Each organ was evaluated on parameters such as inflammatory reaction, amount and type of exudate, as well as transparency of the airsac, congestion, proliferation of the spleen, granuloma formation, and other anomalies. Scores ranged from 0 to 4, where 0 represented the absence of lesions and 4 represented marked lesions. Scoring definitions for each parameter are available in File S3.

To compare scorings statistically, scores for each parameter (inflammatory reaction, exudate, etc.) in each tissue (airsac, lung, etc.) were summed by each dose group (50K, 0.5K, 5M, 50M, and control) and divided by the number of animals in the dose group to obtain weighted scores. By summing all weighted scores from all control samples in both *in vivo* studies and dividing by the total number of control animals (*n* = 42) multiplied by the total number of tested parameters (*n* = 15), a baseline control frequency was obtained. Using a binomial test, this frequency was used to compare summed scorings from individual parameters for each infected dose group, as the number of successes with the total number of animals examined for each parameter in each tissue as the number of trials. Finally, the *P*-values were subjected to multiple testing correction using Benjamini-Hochberg (FDR) correction. Scoring definitions for each parameter are available in File S3.

### Specificity cohort, clinical, and real-world samples

The specificity cohort comprised serum samples (*n* = 24) from two previously conducted longitudinal studies with *E. coli* infection (Bojesen et al. 2026, in prep) and *G. anatis* infection (81) in chickens. Briefly, the *E. coli* group comprised a total of 12 serum samples from six chickens (breed: Ross-308) infected intra-tracheally with 1 × 10⁶ live colony-forming units (CFU) per chicken of *E. coli* (strain DH269, sequence type (ST) 428) in a longitudinal study design: blood draws were conducted on 23 January 2024, immediately before intra-tracheal infection, and from the same individuals on 25 January 2024 or 2 days post-infection (dpi). The *G. anatis* group comprised a total of 12 serum samples from six chickens (breed: 16-week-old Lohmann-Brown layer chickens) infected intra-peritoneally with 10⁷ live CFU per chicken of *G. anatis* (strain: 10,672/9) in a longer longitudinal study design: blood draws were conducted on 16 April 2019, and again from the same individuals on 6 May 2019 or 21 days dpi (81).

Serum samples from clinical cases were kindly shared by Laboklin GmbH diagnostic facility (Bad Kissingen, Germany) and were used as unrelated suspected positive samples. Prior to this study, each sample was tested using an indirect hemagglutination assay (IHA) using the Hemkit *Aspergillus* IHA (Ravo Diagnostika GmbH, Germany). Serum was taken on 28 December 2023, 16 October 2023, and 30 September 2023, with IHA titers of 1:80, 1:160, and 1:320, respectively. Finally, three serum samples from clinically healthy adult chickens were obtained from the Copenhagen Zoo and were used as suspected negative blinded samples. Each of the three samples was taken on 21 January 21, 2010, and serum had been stored undisturbed at −80°C since collection.

### Serum protein electrophoresis—*in vivo* studies and specificity cohort

To assess the acute phase proteins (APPs), the humoral immune response, and general health condition in the *in vivo* chicken models and performance cohort, SPE was conducted to obtain albumin/globulin (A/G) ratios. Total protein in g/dL (TP) was measured in 2 µL serum for each sample using a standard veterinary refractometer (Atago T2-Ne Clinical, Atago Co., Ltd, Japan) at standard atmospheric pressure and room temperature. SPE was conducted for each serum sample by agarose gel electrophoresis (AGE) using the EP001 Kit (DDS Diagnostic S.R.L, Bucharest, Romania) following the manufacturer’s instructions. The gels were loaded and run in a standard Bio-Rad PowerPac Basic gel electrophoresis apparatus (Bio-Rad, CA, USA) at 100V for 20 min. After the fix, stain, and bleach steps, the dried gel was scanned using an Epson V550 (Epson, USA) with SilverFast SE 8.8 (LaserSoft, Kiel, Germany). An electropherogram was generated for each sample using the gel analysis function in ImageJ software (v. 1.54d, NIH, USA (82). Finally, the area under the curve was computed for each of the fractions (prealbumin, albumin, α1, α2, β, and γ), and A:G ratios were calculated as:


$$\text{A:G ratio}=\frac{{\text{Area}}_{\text{prealbumin}}+{\text{Area}}_{\text{albumin}}}{{\text{Area}}_{\alpha }+{\text{Area}}_{\beta }+{\text{Area}}_{\gamma }}$$

as per standard convention (83). To test and assess the inter-assay variation, 10 samples from five animals were analyzed twice, each at different dates. Subsequently, the coefficient of variation (CV) was calculated for TP and A/G ratios as:


$$CV=\frac{SD}{\bar{x}}\times 100\%$$

where SD is the standard deviation and *x̄* is the mean.

### Histopathology and immunohistochemistry on lung tissue

After each necropsy and scoring, one piece of the left lung was fixed in formalin for histology. Briefly, following paraffin embedding, tissue sections of 4 µm in thickness were stained with hematoxylin and eosin (HE). Immunohistochemistry (IHC) was conducted by mounting on positively charged glass slides (Superfrostplus, Thermo Scientific, Germany) and subsequently, the slides were subjected to deparaffinization, rehydration, and antigen retrieval (95°C for 30 min). The immunostaining was conducted with a primary antibody targeting *Af* (Anti-*Aspergillus* Antibody [WF-AF-1] (A281971)) and detected using UltraVision LP Detection System HRP according to the manufacturer’s instructions. The fungal elements of *Af* were stained red. Finally, histopathological evaluations were conducted for 12 parameters for a total of 15 samples from the 50M (*n* = 5) and 5M (*n* = 10) groups in the pilot study, and a total of 20 samples from the 5M (*n* = 10) and control (*n* = 10) groups from the long-term study. Evaluations were compared statistically with non-parametric Wilcoxon tests with Benjamini-Hochberg multiple testing correction (FDR) and visualized using the ggstats (v. 0.7.0) (84) R package.

### Serology-based diagnostics using chicken IgG

To assess *Aspergillus*-specific antibody levels, the LDBio *Aspergillus* IgG kit (LDBio, Lyon, France) western blot kit was used in conjunction with a chicken-IgG conjugate (LDBio, France). All determinations were done according to the manufacturer’s instructions. Positive controls comprised serum obtained from an *Af*-positive penguin confirmed by necropsy, which was collected in January 2023 in the Copenhagen Zoo before euthanasia. The final blots were scanned with an Epson v550 (Epson, USA) using SilverFast SE 8.8 software for confirmation of a minimum of two bands for a positive test in accordance with the manufacturer’s instructions.

### Randomized train-test split and sample allocation

In total, 124 samples were selected for cfDNA ONT sequencing from all studies: long-term study (*n* = 72), pilot study (*n* = 22), specificity cohort (*n* = 24), and clinical and real-world samples (*n* = 6). All samples were pooled regardless of study origin and randomly split into a training set (*n* = 75) and an independent blinded test set (*n* = 49). The randomization was performed using stratified random sampling (random seed: 123) to ensure balanced representation of infected and control samples in both sets, as well as representation of the bacterial-infected specificity cohort samples. The test set was held out and blinded during all model development, hyperparameter tuning, and selection procedures. This randomized approach prevents study-specific bias and provides unbiased estimates of generalization performance across diverse sample sources and confounding variables.

### Cell-free DNA extractions and quality control

A total of 124 serum samples were selected for cfDNA extractions and ONT methylation sequencing. All serum samples had been stored at −80°C immediately after collection and processing. A total of 200 µL serum per sample was used as input for cfDNA extraction with the Norgen Biotek Circulating Cell-Free DNA Micro kit (cat. no. 55500) following the manufacturer’s instructions. Before extractions, all serum samples were subjected to a centrifugation step for 2 min at 400 × *g* (~2,000 RPM) prior to extraction. After cfDNA extraction, all samples were measured on a Bioanalyzer 2100 (Agilent, Germany) using the High Sensitivity DNA system (cat. no. 5067-4626) following the manufacturer’s instructions. All cfDNA samples were stored at −80°C. No cfDNA concentrations were available from the clinical and real-world control groups, as these samples were checked by 2% agarose gel.

### ONT sequencing of cell-free DNA

All ONT sample preparation and flow cells were performed using the newest version 14 chemistry. The DNA libraries for sequencing were prepared using the Native Barcoding Ligation Sequencing kit (SQK-NBD114.24) with a sample input volume of 11 µL cfDNA, including 1 µL diluted control DNA (DCS). The sequencing protocol used was the Native Barcoding gDNA 24 (v14-sqk-nbd114-24-NBE_9169_v114_revQ_15Sep2022). All steps were done according to the manufacturer’s instructions, except for the modification that all bead clean-up steps were multiplied by a factor of 2.5× as recommended by F. Martignano et al. (85). Samples were sequenced with either the Nanopore P2 Solo sequencer or the ONT MinION Sequencer (as shown in the library ID available in File S5. All flow cells, both PromethION and MinION flow cells, were of type R10.4.1, and each individual cell was checked before runs for adequate pore numbers. Raw sequencing data in pod5 format was transferred directly to a Samsung T7 external SSD after each run.

### Key methodological workflow summary

All critical methodological parameters used throughout the analysis pipeline are consolidated here for reference. Basecalling was performed using ONT guppy (v. 6.5.7) (86) in High Accuracy mode with base modifications (cg) enabled. The methylation data were extracted in BED file format from individual sample modBAM file using ONT modkit (87) for MethylSense analysis. The methylation data were counted and summarized using six different region sizes (1 KB to 25 KB), generating six matrices with genome-wide methylated regions shared across all samples with sufficient sequencing coverage (>10×). Differential methylation analysis used a two-stage filtering process: statistical significance (FDR < 0.05) and effect size (absolute median methylation difference ≥ 5%). Nested CV used an outer 10-fold CV loop with an inner fivefold CV loop for hyperparameter tuning, repeated 10 times with different random seeds (base seed: 123, with fold-specific offsets). The train-test split was performed by random allocation of all 124 samples into a training set (*n* = 75) and an independent test set (*n* = 49), with stratification to ensure balanced representation of infected and control samples in both sets. The exact MethylSense parameters for each test are in the model evaluation reports for full reproducibility under “METHYLSENSE ANALYSIS SETTINGS” in the Zenodo repository.

### Basecalling, cell-free DNA read mapping, and extracting methylation data

All basecalling, mapping, and extraction of base modification (methylation) data were done on the national life-science high-performance computer cluster (HPC) Computerome (DTU, Risø, DK) and the Aarhus University Genome DK Cluster (Aarhus, DK). First, ONT reads were transferred to the HPC, and raw pod5 files were basecalled and respective cytosine base modifications (5mC and 5hmC) were computed using the ONT guppy (v. 6.5.7) software in High Accuracy mode, using setting the barcode_kits flag to “SQK-NBD114-24” and using the model file “dna_r10.4.1_e8.2_400bps_modbases_5mc_cg_hac.cfg.” Basecalling was accelerated with an Nvidia Tesla V100 GPU. Subsequently, quality assessment of sequencing runs was evaluated using MinIONQC (v. 1.4.2) (88).

The “NanoporeToBED” pipeline (https://github.com/markusdrag/NanoporeToBED-Pipeline) was used to generate MethylSense-ready BED files from the unaligned BAM files from ONT guppy containing methylation data. Briefly, the pipeline mapped each unaligned BAM file to the Ensembl *Gallus gallus* reference genome (bGalGal1.mat.broiler.GRCg7b rel. 108) (89) using samtools Fastq (v. 1.16) (90) piped together with minimap2 (v. 2.24-r1122) (91) in ONT mode. The aligned BAM files were sorted and indexed using samtools and evaluated using qualimap (v. 2.2.1) (92). Finally, the aligned BAM files with methylation data were used as input for the ONT modkit (v. 0.1.2) pileup software in “cpg combine mods,” which extracted all relevant base modifications (methylation patterns) for each sample and generated BED files for each sample that were ready for input into MethylSense. Full sequencing statistics, as well as sample lists with correct barcodes for demultiplexing the raw Nanopore pod5 libraries from this study, can be found in File S5.

### Creating region size classes from the reference genome

The “GenomeToWindows” (https://github.com/markusdrag/GenomeToWindows) tool was used to generate six individual genome partitions: 1 KB, 5 KB, 10 KB, 15 KB, 20 KB, and 25 KB, and each genome partition was saved as an individual BED file for use in MethylSense. This tool automatically downloaded the reference genome and applied the makeWindows function from the bedtools (v. 2.31.1) (93) software to divide the Ensembl chicken reference genome into sequential non-overlapping genomic regions.

### Normalization, coverage threshold, and computing cell-free DNA regions

The following described DMR- and ML workflow was compiled into the automated software package “MethylSense” (https://github.com/markusdrag/MethylSense). Briefly, MethylSense employed the function methRead from the R package methylKit (v. 1.24.0) (94) for loading BED files into a sample methylObject, which checked for irregularities and finally, normalized for read coverage by their median CpG counts using the methylKit normalizeCoverage() function, and the methylObject was ready for binning the cfDNA into six region size classes. The object was saved as a qs file using the qs R package for quick repeated analysis. The normalized methylation percentages generated earlier in the methylObject were binned into their regional methylation counts using the genome partitions generated previously using the regionCounts() function in methylKit, which was repeated six individual times for each partition (1, 5, 10, 15, 20, and 25 KB). Herein, a filtering step was included that required a minimum coverage of 10× cfDNA reads over each region for each sample for a region to be saved for further statistical analysis.

### Finding *Af*-associated differentially methylated regions

Statistical comparison to find DMRs was performed using a logistic regression, where for each region, the methylation proportion (π) was modeled as a function of infection status. The logistic regression model was specified as:


$$log\left(\frac{\pi }{1-\pi }\right)=\beta_{0}+\beta_{1}\cdot \text{Infection Status}$$

where π represents the proportion of methylated cytosines in a given region, β₀ is the intercept, and β₁ is the coefficient for infection status. For each region, the numbers of methylated (M) and unmethylated (U) cytosines were used as input, and the model tested whether the log-odds of methylation differed significantly between infected and control groups. If the fraction of methylated cytosines was significantly different across infected and control groups, the region was defined as an *Af*-specific DMR. Full details of the procedure can be found in A. Akalin et al. (94). Multiple testing correction was applied using the Benjamini-Hochberg false discovery rate (FDR) procedure. Methylated regions with FDR < 0.05 were considered statistically significant and saved as DMRs.

A two-stage filtering process was applied to select DMRs for ML model development. First, DMRs were required to meet statistical significance (FDR < 0.05). Second, DMRs were required to demonstrate an absolute median methylation difference of at least 5% hypermethylation between infected and control groups. The effect size filter was defined as:


$$|\Delta \pi |=|\text{median}(\pi_{\text{infected}})-\text{median}(\pi_{\text{control}})|\geq 0.05$$

where Δπ represents the absolute difference in median methylation between groups. DMRs passing both statistical (FDR < 0.05) and effect size (|Δπ| ≥ 0.05) criteria were selected as candidate features for machine learning model training. The DMRs were visualized using R packages ggpubR (v. 0.6.0) (95), ggplot2 (v. 3.4.3) (96), ggridges (v. 0.5.6) (97), and Pheatmap (v. 1.0.12) (98).

### Machine learning model screening and selection

After the two-stage filtering, the candidate DMRs and their associated methylation data obtained from a randomly selected training set (*n* = 75) were used as features for training the ML models. After training, all ML models were evaluated using an independent blinded test set (*n* = 49), comprising the remaining samples. All model development and evaluation were conducted using the MethylSense software package (v. 5.13.7) developed in this study. A total of 394 machine learning models were screened in detail across six region sizes (1, 5, 10, 15, 20, and 25 KB) and seven classification algorithms: penalized regression (glmnet), neural networks (nnet), support vector machines (svm), random forests (rf), naive Bayes (nb), k-nearest neighbors (knn), and linear discriminant analysis (lda).

For each algorithm and region size combination, models were trained with varying numbers of DMRs, ranked by statistical significance and effect size. Model selection prioritized diagnostic accuracy, sensitivity, specificity, area under the receiver operating characteristic curve (ROC-AUC), precision-recall area under the curve (PR-AUC), number of DMRs, and parsimony. Models were subsequently evaluated using standard Monte Carlo CV and nested CV to obtain unbiased performance estimates, as described below.

### Monte Carlo and nested cross-validation for performance estimation

MethylSense quantified actual model performance using two distinct strategies: (i) 10-repeat Monte Carlo CV for stringent model evaluation and identification of persistently misclassified samples and (ii) nested CV for unbiased performance estimation. The 10-repeat Monte Carlo CV was used as the primary method for model evaluation and performance estimation as the primary quality control step in this study. In this approach, the full data set was randomly partitioned into a training set (60%, *n* = 75) and an independent test set (40%, *n* = 49) for 10 separate repeats, stratified by infection status to maintain balanced class representation. For each repeat, the model was trained on the training set and evaluated on the hold-out test set. Hyperparameter tuning was performed on the training partition using the caret (v. 6.0-94) (99) package in R with algorithm-specific parameter grids: support vector machines (sigma = 0.01, 0.1; *C* = 0.1, 1), neural networks (size = 3, 5, 10; decay = 0, 0.1, 0.01), random forests (mtry based on feature count; splitrule = gini, extratrees; min.node.size = 1, 5), naive Bayes (usekernel = TRUE; adjust = 1; fL = 0.2, 0.5, 0.8), and penalized regression (tuneLength = 3). For other algorithms (k-nearest neighbors, linear discriminant analysis), default caret tuning with tuneLength = 3 was used. Performance metrics, including accuracy, sensitivity, specificity, ROC-AUC, PR-AUC, F1 score, and Matthews correlation coefficient (MCC), were calculated for each repeat, and mean values with 95% confidence intervals were computed across all 10 repeats. The Monte Carlo CV results (*n* = 490 total evaluations across 10 repeats) represent the primary performance estimates reported in the Results section and were used for model selection and comparison.

Nested cross-validation was used to obtain unbiased estimates of model performance and to quantify stability across data partitions. An outer 10-fold cross-validation loop was used to partition the data set, and within each outer fold, an inner fivefold cross-validation loop was used for hyperparameter tuning. This nested structure prevents information leakage from the test set into model selection and hyperparameter optimization. For each outer fold, the model was trained on 9/10 of the data (optimized via the inner loop) and evaluated on the held-out 1/10. The entire nested CV procedure (outer 10-fold with inner 5-fold) was repeated 10 times with different random seeds (base seed: 123, with fold-specific offsets) to obtain robust performance estimates. Hyperparameter tuning parameters matched those used in the primary analysis, with algorithm-specific parameter grids as described above. Mean accuracy, sensitivity, specificity, ROC-AUC, PR-AUC, and F1 scores were calculated across all outer folds from all 10 repetitions (*n* = 124 unique birds validated), along with 95% confidence intervals calculated using the standard error of the mean. Models with narrow confidence intervals and high mean performance were prioritized for final selection.

### Bootstrap marker stability analysis

To assess the robustness of DMR selection and identify markers that were not dependent on specific training samples, bootstrap-based stability analysis was performed. For each diagnostic test, *B* = 1,000 bootstrap resamples were generated by sampling with replacement from the training set using random seed 123. For each bootstrap resample *b* (*b* = 1, 2, …, *B*), DMRs were re-selected using the same two-stage filtering criteria (FDR < 0.05 and absolute methylation difference ≥5%), and the selection frequency for each DMR was calculated as:


$$f_{i}=\frac{1}{B}\sum_{b=1}^{B}I({DMR}_{i}\;\text{selected in bootstrap }b)$$

where I(·) is an indicator function equal to 1 if DMR *i* was selected in bootstrap resample *b*, and 0 otherwise. DMRs with selection frequency *fᵢ* ≥ 0.75 (selected in at least 75% of bootstrap resamples) were considered highly stable. The 75% threshold was selected to identify markers with high consistency across bootstrap resamples, balancing stringency with practical marker selection. This analysis was performed separately for each of the three final diagnostic tests to assess marker stability.

### Batch effects and technical covariate assessment

To assess whether diagnostic performance was driven by biological signal rather than technical artifacts, formal testing for batch effects and technical confounding was performed. For each DMR in each diagnostic test, linear models were fitted that included infection status as the primary predictor alongside 13 technical and pre-analytical covariates: sequencer (P2 Solo vs MinION), cfDNA concentration, number of reads, mapping percentage, mean coverage, total protein, albumin/globulin ratio, and serum protein fractions (pre-albumin, albumin, alpha-1 globulin, alpha-2 globulin, beta globulin, and gamma globulin). The linear model for each DMR was specified as:


$$\pi_{i}=\alpha +\gamma \cdot \text{Infection Status}+\sum_{j=1}^{13}\delta_{j}\cdot X_{j}+\epsilon_{i}$$

where π*ᵢ* is the methylation proportion for DMR *i*, α is the intercept, γ is the coefficient for infection status, δ*ⱼ* are coefficients for the 13 technical covariates X*ⱼ*, and ε*ᵢ* is the error term. Models were fitted using the training set, and associations between DMR methylation and each covariate were tested after adjusting for infection status. The null hypothesis H₀: δ*ⱼ* = 0 was tested for each covariate *j*, and *P*-values were corrected for multiple testing using Benjamini-Hochberg FDR correction with a threshold of FDR < 0.05. FDR correction was applied globally across all DMRs and all covariates for each diagnostic test. DMRs with significant covariate associations after FDR correction (FDR < 0.05) were identified, and the number of such DMRs was reported for each covariate and each diagnostic test. Principal component analysis (PCA) and uniform manifold approximation and projection (UMAP) were performed to visualize sample clustering by technical covariates versus infection status, using the R packages factoextra (v. 1.0.7) (100) and uwot (v. 0.1.16) (101).

### Temporal methylation dynamics analysis

To assess whether DMR methylation changed over time during the course of infection, temporal dynamics were examined by modeling methylation trajectories across sampling weeks. For the long-term study samples, methylation values for each DMR were compared across different time points (week 2, week 4, week 5) using Kruskal-Wallis tests, which are non-parametric and robust to non-normal distributions. The Kruskal-Wallis test statistic *H* was calculated as:


$$H=\frac{12}{N(N+1)}\sum_{i=1}^{k}\frac{R_{i}^{2}}{n_{i}}-3(N+1)$$

where *k* is the number of time points (*k* = 3), *N* is the total number of observations, *nᵢ* is the number of observations at time point *i*, and *Rᵢ* is the sum of ranks for time point *i*. Under the null hypothesis of no difference across time points, *H* follows a chi-square distribution with *k* − 1 degrees of freedom. *P*-values were corrected for multiple testing using Benjamini-Hochberg FDR correction with a threshold of FDR < 0.05. DMRs with adjusted *P* < 0.05 (FDR < 0.05) were considered to show significant time-dependent changes. For each significant DMR, the direction of change (increasing, decreasing, or stable with variation) was determined by comparing median methylation values across time points. This analysis was performed separately for each of the three diagnostic tests to assess the temporal stability of the DMR panels.

### WGCNA co-methylation network analysis

Weighted gene co-expression network analysis (WGCNA) was performed to identify co-methylated modules of DMRs that may reflect coordinated epigenetic regulation. The WGCNA R package (v. 1.72-1) (102) was used to construct signed correlation networks from DMR methylation data in the training set. A soft-thresholding power was selected to achieve scale-free topology, and modules were identified using hierarchical clustering with dynamic tree cutting. Module-trait relationships were assessed by correlating module eigengenes (the first principal component of each module) with infection status. Modules showing strong positive or negative correlations with infection were identified, and DMRs within these modules were examined for overlap with high-effect-size and stable DMRs identified in other analyses.

### Final selection of the three diagnostic tests

All machine learning models that passed the nested CV screening were evaluated on the independent blinded test set (*n* = 49). Final selection of the three diagnostic tests was based on prioritized criteria: (i) diagnostic accuracy on the test set, (ii) sensitivity and specificity for detection of *Aspergillus* infection, (iii) ROC-AUC and PR-AUC values, (iv) number of DMRs (prioritizing parsimony), (v) region size class of the DMRs, (vi) marker stability from bootstrap analysis, (vii) absence of batch effects, and (viii) performance across different study cohorts (pilot study, long-term study, specificity cohort, clinical, and real-world samples). Three final diagnostic tests were selected: the High Accuracy test (93 DMRs, neural network classifier), the Fast test (35 DMRs, support vector machine classifier), and the *In Situ* test (5 DMRs, random forest classifier).

### Individual DMR importance and functional annotations

Summary statistics, chromosome mapping, genomic locations, and functional annotation were conducted with the R packages BRGenomics (v. 1.12.0) (103) and genomation (v. 1.36.0) (104). The contribution of individual DMRs to group separation was quantified using ANOVA effect sizes (eta squared, η²) calculated from one-way analysis of variance models comparing methylation between infected and control groups. For each DMR, a one-way ANOVA was fitted:


$$\pi_{ij}=\mu +\alpha_{j}+\epsilon_{ij}$$

where π*ᵢⱼ* is the methylation proportion for sample *i* in group *j* (*j* = infected or control), μ is the overall mean, α*ⱼ* is the group effect, and ε*ᵢⱼ* is the error term. Eta squared was calculated as:


$$\eta^{2}=\frac{{SS}_{\text{between}}}{{SS}_{\text{total}}}=\frac{\sum_{j}n_{j}{\left({\overset{‾}{\pi }}_{j}-\overset{‾}{\pi }\right)}^{2}}{\sum_{i,j}{\left(\pi_{ij}-\overset{‾}{\pi }\right)}^{2}}$$

where SS_between_ is the sum of squares between groups, SS_total_ is the total sum of squares, *nⱼ* is the number of samples in group *j*, π̄*ⱼ* is the mean methylation in group *j*, and π̄ is the overall mean. Eta squared values were interpreted as follows: negligible (η² < 0.01), small (0.01 ≤ η² < 0.06), medium (0.06 ≤ η² < 0.14), or large (η² ≥ 0.14). Function annotation of features such as genes, transcripts, and exons was conducted using the Ensembl chicken feature genome (bGalGal1.mat.broiler.GRCg7b release 108). For regulatory features and Epigenetically Modified Accessible Regions (EMARs), the Ensembl chicken regulatory features reference genome was used (bGalGal1.mat.broiler.GRCg7b release 113). Functional analysis revealed that DMRs in all three diagnostic tests overlapped regulatory and genic elements, including genes, transcripts, exons, promoters, enhancers, EMARs, and open chromatin regions.

### Reference methylation values and infection probabilities for each diagnostic test

Reference values for each diagnostic test (High Accuracy test [*n* = 93 DMRs], Fast test [*n* = 35 DMRs], *In Situ* test [*n* = 5 DMRs]) were calculated by logistic regression using a binomial generalized linear model (glm) in R. For each test, the median methylation value across all DMRs in the panel was calculated per sample as:


$$\pi_{\text{median}}=\text{median}(\pi_{1},\pi_{2},...,\pi_{k})$$

where *k* is the number of DMRs in the panel (*k* = 93, 35, or 5) and π*ᵢ* represents the methylation proportion for DMR *i*. Logistic regression was used to relate median panel methylation to the probability of *Aspergillus* infection:


$$log\left(\frac{P(\text{Infection})}{1-P(\text{Infection})}\right)=\beta_{0}+\beta_{1}\cdot \pi_{\text{median}}$$

which can be rearranged to express the infection probability as:


$$P(\text{Infection})=\frac{1}{1+e^{-(\beta_{0}+\beta_{1}\cdot \pi_{\text{median}})}}$$

where β₀ is the intercept and β₁ is the coefficient for median methylation. Using the fitted logistic regression models, reference methylation values and intervals for clinical interpretation of new samples without running the full machine learning models were calculated for each of the three diagnostic tests for infection probabilities of 0.01, 0.05, 0.10, 0.50, 0.90, 0.95, and 0.99.

### PCR primer preparation for *In Situ* diagnostic test

To support independent validation and potential clinical deployment of the *In Situ* diagnostic test, targeted PCR resources were prepared for all five constituent DMRs. For each DMR, core and extended genomic coordinates were defined based on the Ensembl chicken reference genome (bGalGal1.mat.broiler.GRCg7b release 108). Direct links were generated to the UCSC Genome Browser (https://genome.ucsc.edu/) for visualization, sequence retrieval, and contextual inspection of each DMR. Preconfigured links were also generated to the MethPrimer webserver (http://www.urogene.org/methprimer/) for bisulfite-specific primer design. Recommended design parameters were standardized across all five DMRs: primer length of 18–27 bases, expected product size of 100–500 base pairs, and avoidance of CpG dinucleotides within primer-binding sites. All five regions fall within the sequence length limits of commonly used bisulfite primer design tools. These resources enable straightforward development of bisulfite PCR, pyrosequencing, methylation-specific PCR, or high-resolution melt assays targeting the *In Situ* DMRs.

## Data Availability

Preloaded models for the High Accuracy, Fast, and In Situ tests with all necessary DMR data are available in the Zenodo repository, enabling the prediction of Af status in new, unknown chicken samples using MethylSense predict. The repository also contains methylation files for each sample (n = 124) in bed format and in .qs format for flexible and easy loading into MethylSense. DOI: 10.5281/zenodo.15194045 All ONT raw sequencing data in pod5 libraries supporting the findings are available through the European Bioinformatics Institute (EBI), European Nucleotide Archive (ENA): Accession no.: PRJEB86769 For basecalling and demultiplexing the libraries, sample barcode lists can be found in File S5.

## SUPPLEMENTAL MATERIAL

File S1 jcm.01054-25-s0001.docx Dose quality control.

File S2 jcm.01054-25-s0002.pdf Postmortem scores plus immunohistochemistry data.

File S3 jcm.01054-25-s0003.docx Postmortem scoring sheet with scoring grading definitions for each individual parameter.

File S4 jcm.01054-25-s0004.docx Cell free DNA conc.

File S5 jcm.01054-25-s0005.xlsx Sequence data stats.

File S6 jcm.01054-25-s0006.pdf General methylation data landscape.

File S7 jcm.01054-25-s0007.xlsx Sample inconsistencies.

ASM does not own the copyrights to Supplemental Material that may be linked to, or accessed through, an article. The authors have granted ASM a non-exclusive, world-wide license to publish the Supplemental Material files. Please contact the corresponding author directly for reuse.
