# Supplementary material for: MethylSense: high accuracy machine learning-based diagnostics for Aspergillus fumigatus infection in chickens using host cell-free DNA methylation and Nanopore sequencing
Source: J Clin Microbiol. 2026 Apr 27;64(6):e01054-25. doi: 10.1128/jcm.01054-25 (PMC13251387; doi:10.1128/jcm.01054-25)
Supplement: File S2 — Postmortem scores plus immunohistochemistry data. [file jcm.01054-25-s0002.pdf]

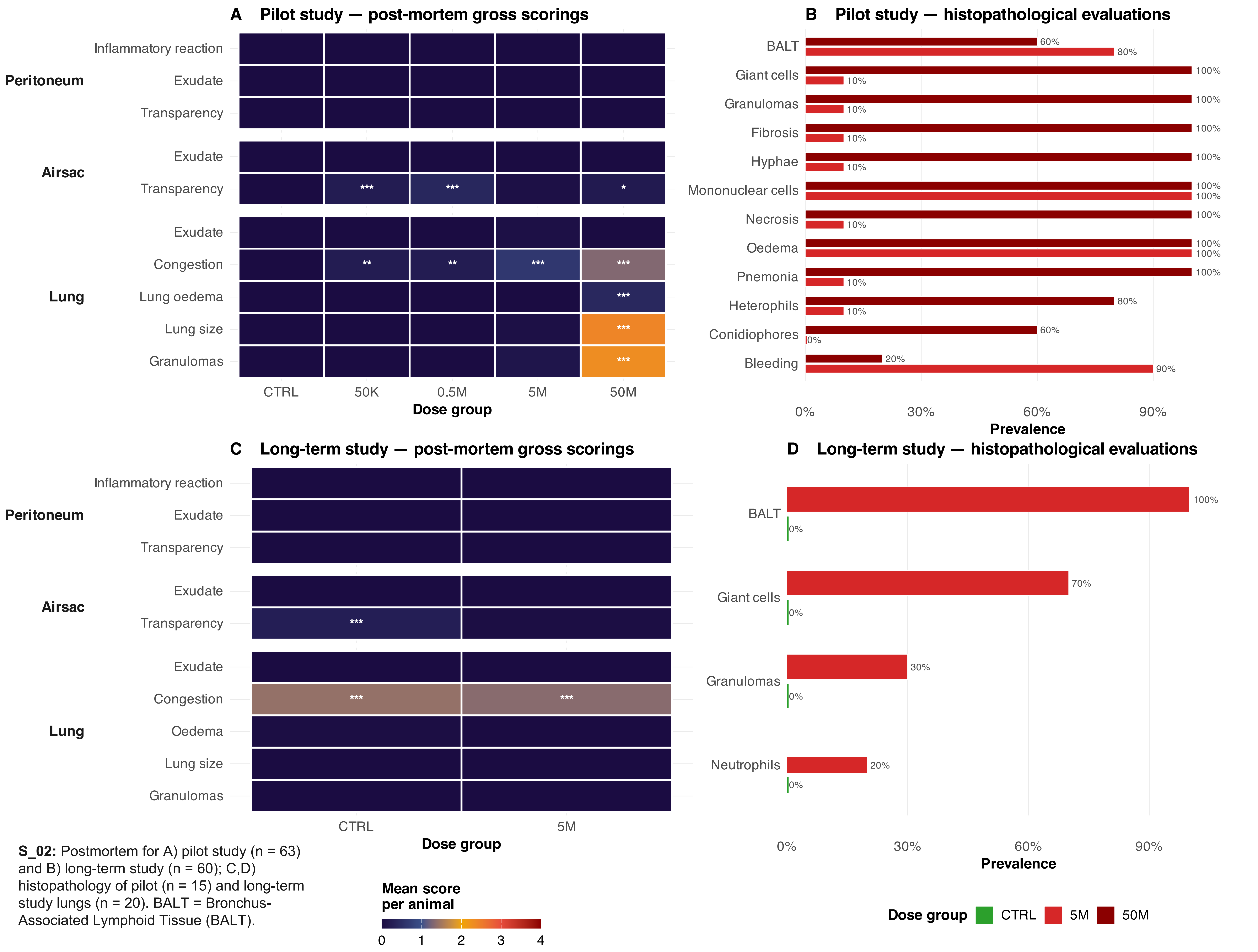

S_02: Postmortem for A) pilot study (n = 63) and B) long-term study (n = 60); C,D) histopathology of pilot (n = 15) and long-term study lungs (n = 20). BALT = Bronchus-Associated Lymphoid Tissue (BALT).
