# Supplementary material for: MethylSense: high accuracy machine learning-based diagnostics for Aspergillus fumigatus infection in chickens using host cell-free DNA methylation and Nanopore sequencing
Source: J Clin Microbiol. 2026 Apr 27;64(6):e01054-25. doi: 10.1128/jcm.01054-25 (PMC13251387; doi:10.1128/jcm.01054-25)
Supplement: File S4 — Cell free DNA conc. [file jcm.01054-25-s0004.docx]

**S_04:** Cell-free DNA concentrations and sequencing statistics for all runs.

**Table 1: Mean cell-free DNA concentrations and sequencing reads obtained from the long-term study.**

| **Long-term study** |  | **Avg. cell-free DNA (± SD)**  **(ng/µl per animal)** | | **Avg. reads (± SD)**  **(million reads/animal)** | | **ONT device** |
| --- | --- | --- | --- | --- | --- | --- |
|  | **n** | **Control** | **Infected** | **Control** | **Infected** |  |
| Week 2 | 24 | 5.9  (± 4.1) | 6.3  (± 2.0) | 9.2M  (± 3.2M) | 10.2M  (± 1.8M) | P2 Solo |
| Week 4 | 24 | 12.8  (± 20.4) | 4.9  (± 2.9) | 13.1M  (± 3.1M) | 8.3M  (± 3.6M) | P2 Solo |
| Week 5 | 24 | 88.4  (± 110.3) | 9.0  (± 3.4) | 8.4M  (± 2.7M) | 10.0M  (± 3.0M) | P2 Solo |
| *Sum / mean* | *72* | 57.5  (± 48.7) | 7.4  (± 2.8) | 10.1M  (± 3.1M) | 9.6M  (± 2.8M) |  |

ONT: Oxford Nanopore sequencing; SD: standard deviation.

**Table 2:** **Mean cell-free DNA concentrations and sequencing reads obtained from the four evaluation cohorts.**

| **Evaluation cohorts** |  | **Avg. cell-free DNA (± SD)**  **(ng/µl per animal)** | | **Avg. reads (± SD)**  **(million reads/animal)** | | **ONT device** |
| --- | --- | --- | --- | --- | --- | --- |
|  | **n** | **Control** | **Infected** | **Control** | **Infected** |  |
| Pilot study | 22 | 28.2  (± 19.2) | 19.0  (± 15.5) | 1.6M  (± 0.26M) | 1.7M  (± 0.8M) | MinION |
| Specificity cohort -  *E. coli* | 12 | 4.0  (± 1.5) | 7.2  (± 3.1) | 4.7M  (± 1.3M) | 6.5M  (± 1.3M) | P2 Solo |
| Specificity cohort -  *G. anatis* | 12 | 9.0  (± 4.6) | 13.0  (± 2.2) | 5.0M  (± 1.9M) | 6.0M  (± 0.4M) | P2 Solo |
| Clinical & real-world samples | 6 | † *gel* | 3.8  (± 2.4) | 2.0M  (± 1.4M) | 1.5M  (± 1.3M) | MinION |
| *Sum / mean* | 52 | 13.7  (± 8.4) | 10.7  (± 5.8) | 3.4M  (± 1.2M) | 3.9M  (± 0.9M) |  |

† = samples were analysed with 2% agarose gel.
