# Supplementary material for: MethylSense: high accuracy machine learning-based diagnostics for Aspergillus fumigatus infection in chickens using host cell-free DNA methylation and Nanopore sequencing
Source: J Clin Microbiol. 2026 Apr 27;64(6):e01054-25. doi: 10.1128/jcm.01054-25 (PMC13251387; doi:10.1128/jcm.01054-25)
Supplement: File S6 — General methylation data landscape. [file jcm.01054-25-s0006.pdf]

S_06: General methylation landscape report for all samples (n = 124) in this study from MethylSense.

MethylSense: General Methylation Statistics Report

Analysis Date: 2025-12-02 **Script Version**: 5.13.7 **Generated by**: MethylSense DMR Landscape Analysis Pipeline

---

Executive Summary

This report presents a comprehensive landscape analysis of differential methylation across **6 region sizes** (1 KB, 5 KB, 10 KB, 15 KB, 20 KB, 25 KB). Analysis identified a total of **22658 significant DMRs** (FDR < 0.05) across all region sizes, with detection rates ranging from **27.1% to 47.3%**.

Table of Contents

1. DMR Detection Rates
2. Effect Size Analysis
3. Coverage Quality Control
4. Chromosomal Distribution
5. Methylation Patterns
6. Sample-Level Clustering
7. Statistical Summary

---

1. DMR Detection Rates

Overview

1 KB regions: - Total methylation regions (MRs): **74** - Significant DMRs (FDR < 0.05): **35** - Detection rate: **47.3%**

5 KB regions: - Total methylation regions (MRs): **1,535** - Significant DMRs (FDR < 0.05): **514** - Detection rate: **33.5%**

10 KB regions: - Total methylation regions (MRs): **10,594** - Significant DMRs (FDR < 0.05): **3,077** - Detection rate: **29.0%**

15 KB regions: - Total methylation regions (MRs): **19,269** - Significant DMRs (FDR < 0.05): **5,289** - Detection rate: **27.4%**

20 KB regions: - Total methylation regions (MRs): **24,052** - Significant DMRs (FDR < 0.05): **6,521** - Detection rate: **27.1%**

25 KB regions: - Total methylation regions (MRs): **25,613** - Significant DMRs (FDR < 0.05): **7,222** - Detection rate: **28.2%**

Figure 1: DMR Counts by Region Size

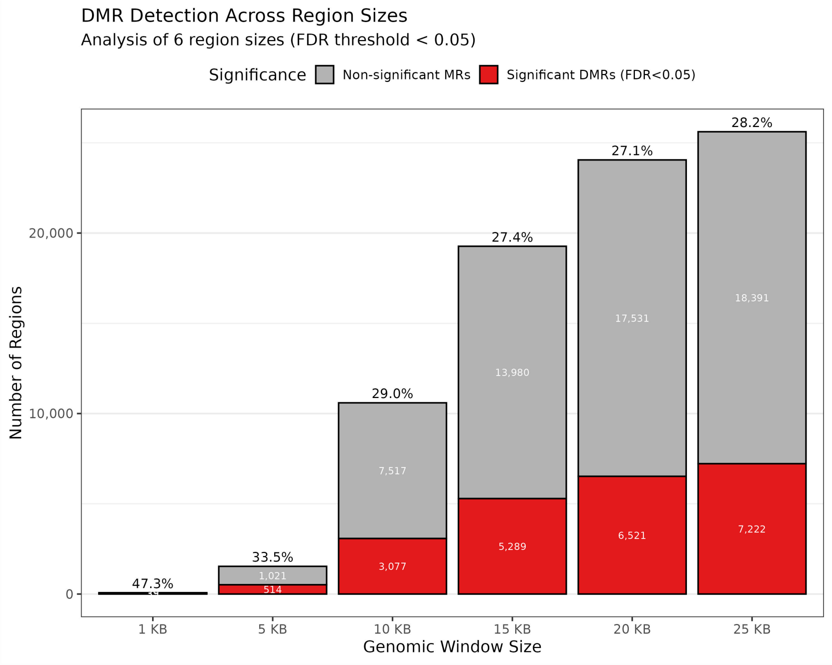

Bar plot showing the number of significant DMRs (FDR < 0.05) vs non-significant MRs across different genomic region sizes. Numbers on bars indicate exact counts. Larger region sizes generally show higher detection rates.

---

2. Effect Size Analysis

Methylation Direction (1 KB regions)

Hypermethylated DMRs: 34 (97.1%) - Mean effect size: **Δβ = +3.05%** (SD = 1.83%) - Median effect size: **Δβ = +2.77%** (IQR: 1.70% to 3.70%)

Hypomethylated DMRs: 1 (2.9%) - Mean effect size: **Δβ = -2.17%** (SD = NA%) - Median effect size: **Δβ = -2.17%** (IQR: -2.17% to -2.17%)

Figure 2: Volcano Plots

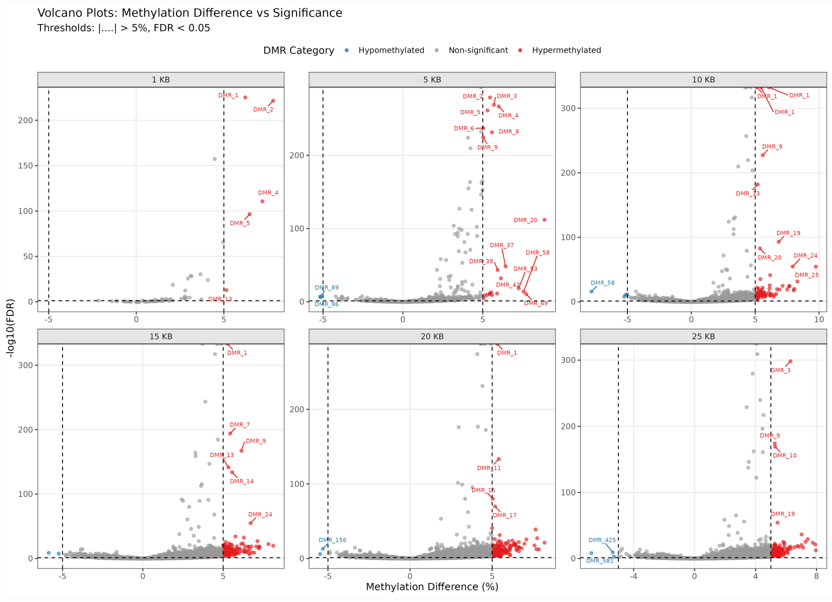

Volcano plots showing methylation difference (Δβ) vs statistical significance ($-\log_{10}$ FDR) for each region size. Horizontal dashed line: FDR threshold (0.05). Vertical dashed lines: effect size threshold (±5.0%). Top 100 DMRs are labelled by rank. Points coloured by direction: red (hypermethylated), blue (hypomethylated).

Figure 3: Effect Size Distribution

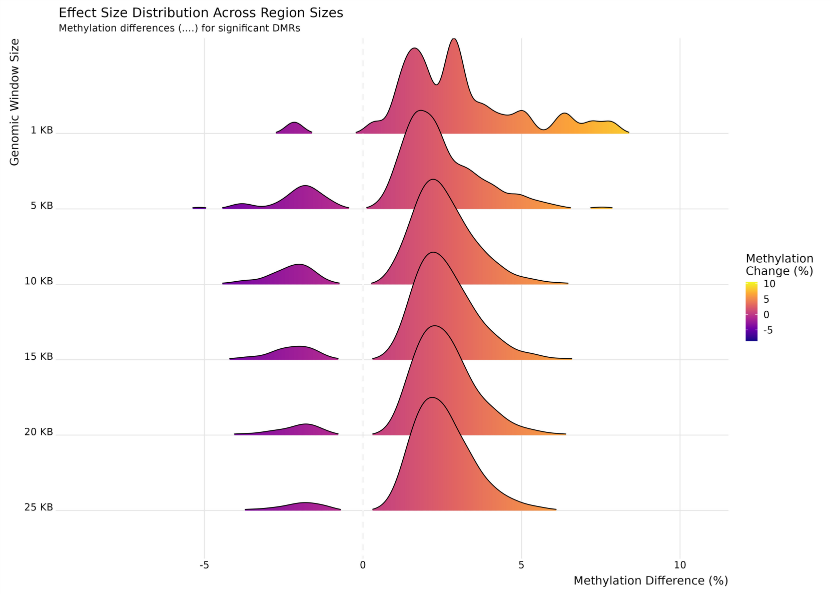

Ridge plot showing the distribution of methylation effect sizes (Δβ) across different region sizes. Colour gradient represents effect size magnitude. Separate distributions shown for hypermethylated (positive) and hypomethylated (negative) DMRs.

---

3. Coverage Quality Control

Coverage Statistics

DMR Coverage: - Median: **115.9×** (IQR: 106.5–136.2×) - Mean: **123.5×** (SD: 22.7×) - Range: 103.1–159.9× across region sizes

Non-DMR Coverage: - Median: **147.3×** (IQR: 143.2–151.3×) - Mean: **161.4×** (SD: 37.4×) - Range: 142.2–237.4× across region sizes

Figure 4: Coverage Distribution

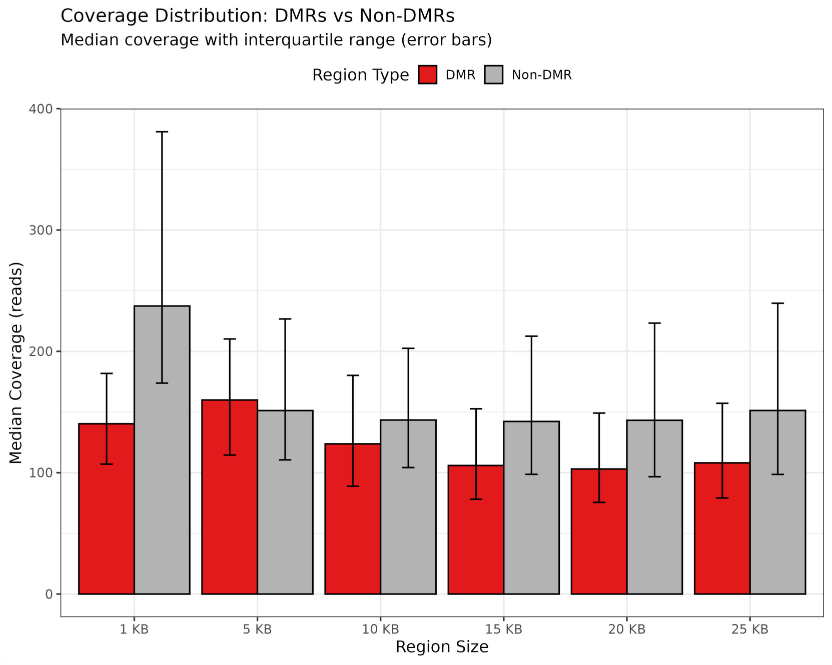

Box plots comparing sequencing coverage between DMRs and non-DMRs across different region sizes. Higher coverage in DMRs indicates better statistical power for differential methylation detection.

---

4. Chromosomal Distribution

Figure 5: DMR Distribution Across Chromosomes

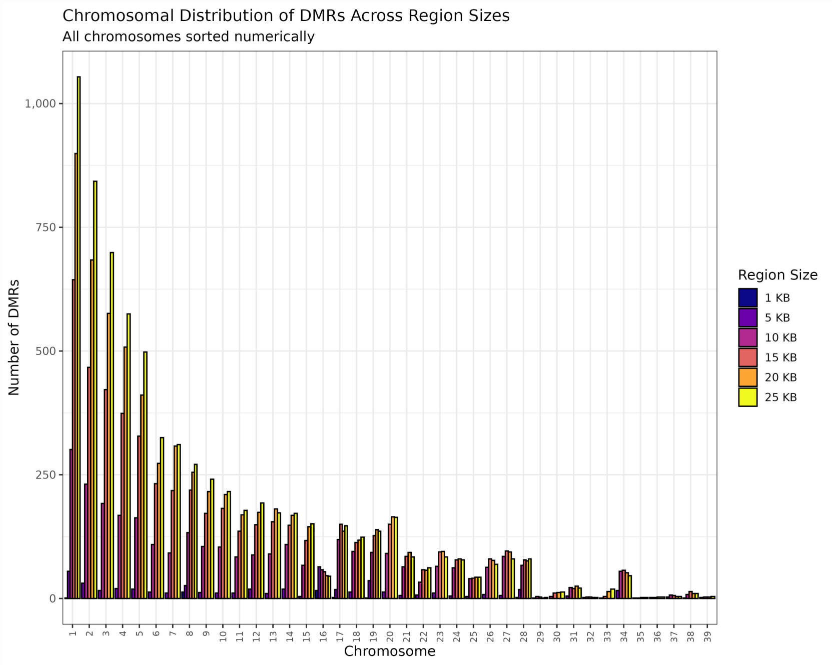

Bar plot showing the distribution of DMRs across all chromosomes (sorted numerically). Different colours represent different region sizes. Identifies chromosomes with DMR hotspots.

---

5. Methylation Patterns by Infection Status

Figure 6: Methylation Distribution Ridge Plot

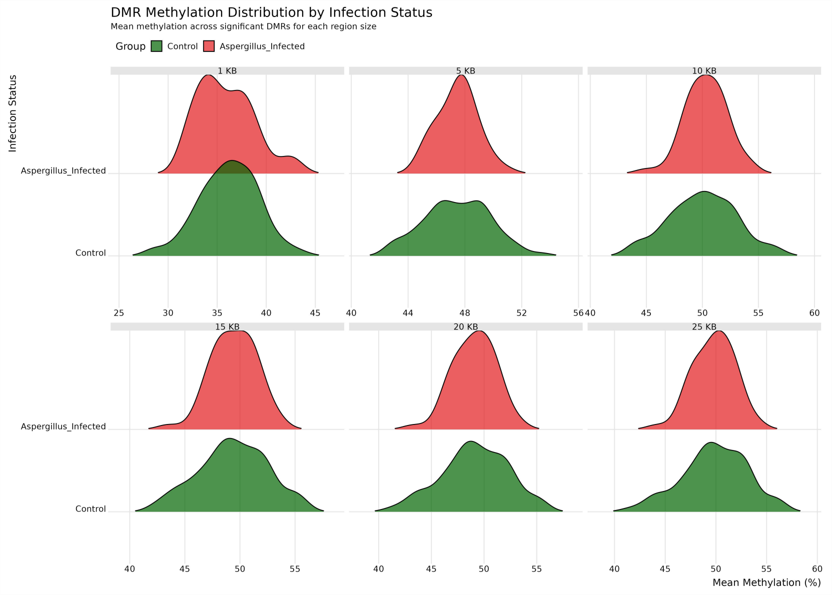

Ridge plots showing methylation percentage distributions for Control vs Infected samples across all region sizes. Each facet represents one region size with Control (green) and Infected (red) distributions stacked vertically. Reveals systematic methylation differences between groups and how separation changes with region size.

---

6. Sample-Level Clustering

Heatmaps were generated for the top 100 DMRs in each region size, showing methylation patterns across all samples. Samples are clustered hierarchically using Euclidean distance and complete linkage.

Heatmap Files

Heatmaps show methylation percentage (0-100%) for top 100 DMRs ranked by FDR. Columns represent samples (annotated by infection status and study). Rows represent DMRs (ordered by hierarchical clustering). Colour annotation: Control (dark green), Infected (red), Study (ColorBrewer Set2 palette).

---

7. Statistical Summary

Summary Table: DMR Counts by Region Size

Region Size	Total MRs	Significant DMRs	Detection Rate
1 KB	74	35	47.3%
5 KB	1,535	514	33.5%
10 KB	10,594	3,077	29.0%
15 KB	19,269	5,289	27.4%
20 KB	24,052	6,521	27.1%
25 KB	25,613	7,222	28.2%

Data Files

All statistical summaries are available in the `stats/` directory:

- **dmr_counts_summary.csv**: Region size, total MRs, significant DMRs, detection rates
- **volcano_plot_stats.csv**: Counts by quadrant (significant hyper/hypo, non-significant)
- **coverage_distribution.csv**: Median, IQR, mean, SD for DMRs and non-DMRs
- **dmr_chromosome_distribution.csv**: DMR counts per chromosome for each region size
- **effect_size_distribution.csv**: Mean, median, min, max effect sizes by direction

---

Methods Summary

Differential Methylation Analysis

Differential methylation analysis was performed using methylKit across 6 genomic region sizes (1 KB, 5 KB, 10 KB, 15 KB, 20 KB, 25 KB). Regions with FDR < 0.05 were considered significant DMRs. Effect sizes represent absolute methylation difference ($\Delta\beta$) between groups, with a threshold of ±5.0% used for biological significance. Coverage quality control ensured median coverage of 115.9× for DMRs and 147.3× for non-DMRs.

Visualization

All plots were generated using ggplot2 (bar plots, volcano plots, box plots), ggridges (ridge plots), and pheatmap (heatmaps). Figures were exported as JPG, SVG format at 300 DPI with dimensions 12 × 8 inches.

Conclusions

This landscape analysis identified **22658 significant DMRs** across 6 region sizes, with detection rates ranging from 27.1% to 47.3%. The majority of DMRs showed hypermethylation (97.1%) with mean effect size of +3.05%, while hypomethylated regions (2.9%) showed mean effect size of -2.17%. Chromosomal distribution revealed DMR hotspots on specific chromosomes, and sample-level clustering confirmed distinct methylation patterns between infection status groups. Coverage quality was consistent across region sizes, ensuring robust statistical inference.

Report generated: 2025-12-02 13:59:29 **Script version**: 5.13.7 **Output directory**:
